# *Psidium guajava* L. An Incalculable but Underexplored Food Crop: Its Phytochemistry, Ethnopharmacology, and Industrial Applications

**DOI:** 10.3390/molecules27207016

**Published:** 2022-10-18

**Authors:** Muhammad Imran Tousif, Mamona Nazir, Muhammad Saleem, Saba Tauseef, Nusrat Shafiq, Laiba Hassan, Hidayat Hussian, Domenico Montesano, Daniele Naviglio, Gokhan Zengin, Ishtiaq Ahmad

**Affiliations:** 1 Department of Chemistry, Division of Science and Technology, University of Education, Lahore 54000, Pakistan; 2 Department of Chemistry, Govt. Sadiq College Women University Bahawalpur, Bahawalpur 63100, Pakistan; 3 Division of Organic Chemistry, Institute of Chemistry, Baghdad-ul-Jadeed Campus, The Islamia University of Bahawalpur, Bahawalpur 63100, Pakistan; 4 Dr. Panjwani Center for Molecular Medicine and Drug Research, International Center for Chemical and Biological Sciences, University of Karachi, Karachi 75270, Pakistan; 5 Department of Chemistry, Government College Women University Faisalabad, Faisalabad 38000, Pakistan; 6 Department of Pharmacy, Faculty of Pharmacy, The Islamia University of Bahawalpur, Bahawalpur 63100, Pakistan; 7 Department of Bioorganic Chemistry, Leibniz Institute of Plant Biochemistry, Weinberg 3, D-06120 Halle, Germany or; 8 Department of Pharmacy, University of Naples Federico II, Via D. Montesano 49, 80131 Naples, Italy; 9 Department of Chemical Sciences, University of Naples Federico II, Via Cintia, 4, 80126 Naples, Italy; 10 Department of Biology, Science Faculty, Selcuk University, Konya 42130, Turkey; 11 Department of Chemical Engineering and Biotechnology, University of Cambridge, Philipa Fawcett Drive, Cambridge CB3 0AS, UK

**Keywords:** *Psidium guajava* L., phytochemistry, meroterpenoid, pharmacological activities, industrial application

## Abstract

*Psidium guajava* L. (guava) is a small tree known for its fruit flavor that is cultivated almost around the globe in tropical areas. Its fruit is amazingly rich in antioxidants, vitamin C, potassium, and dietary fiber. In different parts of the world, this plant holds a special place with respect to fruit and nutritional items. Pharmacological research has shown that this plant has more potential than just a fruit source; it also has beneficial effects against a variety of chronic diseases due to its rich nutritional and phytochemical profile. The primary goal of this document is to provide an updated overview of *Psidium guajava* L. and its bioactive secondary metabolites, as well as their availability for further study, with a focus on the health benefits and potential industrial applications. There have been several studies conducted on *Psidium guajava* L. in relation to its use in the pharmaceutical industry. However, its clinical efficacy and applications are still debatable. Therefore, in this review a detailed study with respect to phytochemistry of the plant through modern instruments such as GC and LC-MS has been discussed. The biological activities of secondary metabolites isolated from this plant have been extensively discussed. In order to perform long-term clinical trials to learn more about their effectiveness as drugs and applications for various health benefits, a structure activity relationship has been established. Based on the literature, it is concluded that this plant has a wide variety of biopharmaceutical applications. As a whole, this article calls for long-term clinical trials to obtain a greater understanding of how it can be used to treat different diseases.

## 1. Introduction

The scientific community’s interest in plant growing is particularly with regard to the chemical components of bioactive compounds, their effect on pathogens and their application as functional foods and/or nutraceuticals to human health [1]. In addition, as various food sources, plant products provide several health benefits. *Psidium guajava* L., commonly known as guava is a small tree, and is grown in tropical areas of world due to its fruits. Guava leaf tea and some complementary items are available in many stores in Japan [2], as it is considered that phenolic compounds of guava leaves can resolve particular health issues such as the modulation of blood sugar levels [3]. In addition to being a healthy and tasty food, the fruit is an excellent source of dietary fiber. Eating guavas may aid healthy bowel movements and prevent constipation [4]. One guava per day can provide 12% of the recommended daily intake of fiber [5]. Additionally, *P.guajava* L. has many applications which make this plant very important from scientific point of view. Thus, a review covering the literature of recent 7 years has been accomplished. For this purpose, a comprehensive literature survey from 2015 to 2021 was performed with respect to its health benefits, GCMS and LC-MS based phytochemical profiling and bioactive secondary metabolites isolated from the different parts of the plant. The pharmacological activities of various plant parts are also discussed in this article. Further prior reviews of this amazing plant do not describe the plant’s full potential, and hence the gaps left by these reviews are filled by the present article where all-important aspects of *P. guajava* are describe comprehensively.

### 1.1. Review Methodology

The scientific literature has been extensively investigated by the use of Preferred Reporting Items for Systematic Reviews and Meta-Analyses (PRISMA) guidelines. Databases have been searched from 2015 to 2020, including Ovid (Books, Journals, Cochrane, AMED, Embase, and MEDLINE), Scopus, Google Scholar, PubMed and Science Direct. To ensure the inclusion of corresponding works, search terminologies were “*Psidium guajava* L.”, “chemical profiling through GCMS and LC-MS”, “essential oils and GCMS analysis”, “phytochemicals”, “meroterpenoids”. Prime research was carried out for *Psidium guajava* L. It was later joined by pharmaceutical or biological activity; medicinal use or traditional or toxicity or cytotoxicity. To combine the search terms, Boolean operators were used. The literature was also searched manually using a backward and forward approach to the investigation of the gray field.

### 1.2. Morphology of P. guajava L.

*P. guajava* L. of the plant family Myrtaceae belongs to the genus *Psidium*, which consists of about 150 species. The *P. guajava* L. (guava) is the most important member of this genus [6]. History revealed that this plant is originated from southern Mexico or Central America [7]. It was believed that Spanish and Portuguese took this fruit to other parts of the world. Its adaptability to different environments in the world is important factor which makes it worldwide fruit in tropical and sub-tropical regions of the globe. The subcontinent, especially, is a great home for this fruit. Guava plant is an evergreen shrub or tree with 3–10 m height. Its leaves are 4–10 mm long and oval in shape. The flowers are pure white, having five petals and long, multiple central stamens. Its fruit is a berry and medium to large in size with a weight around 100–250 g. Fruit shape may be spherical, ovoid or pyriform depending on the type of plant. Skin of the fresh fruit is dark green which changes to light green-yellow, pale yellow and pure yellow based on the cultivar. The aroma of the fruit is pleasant, and a seed cavity is present in the center of the fruit. The fruit pulp is soft and white to red in color depending upon the type of cultivar.

### 1.3. Nutritional Assessment and Traditional Uses of P. guajava L.

*P. guajava* is cultivated as fruit crop and as medicinal plant. Its fruit has economic significance due to pleasant aroma and taste and is used in production of juice, nectar, paste, jam, jelly and candy bars. The nutritional composition of different parts of guava is given in Table 1, which indicates the high value of the fruit and seeds. Further, the fruit and leaves have great medicinal importance and are used to treat various aliments associated with the stomach. Its leaf extracts have strong antibacterial effect [8]. Guava plant is considered to be an excellent source of ascorbic acids, phenolic compounds and carotenoids with major role in the prevention of most the chronic diseases. Crude fiber in guava is 30.9%, which makes it a good source of antioxidant dietary fiber.

The major uses of the main producing countries for guava leaves are for treatment of diabetes mellitus, cardiovascular diseases, cancer and parasitic infections [4]. The application of the treatment is either oral or topical, depending on the disease. In India, China, Pakistan, and Bangladesh, ingestion through decoction, infusion and boiled preparations is the most common way to resolve many illnesses, such as rheumatism, diarrhea, gastrointestinal problems, diabetes mellitus, infections, inflammatory disorders and cough [8,9,10,11]. The decoction of leaves is used for mouth ulcers in Southeast Asia [8,10,11]. Poultice is externally used in Mexico, Brazil, Philippines, and Nigeria for skin and wound applications. In addition, in Nigeria, it is anti-bactericidal agent and chewing sticks are used for oral treatment [8,9,10,11].

**Table 1:** Nutritional composition of different parts of *P. guajava* L.

The Major Nutritional Composition of *P. guajava* L. Whole Fruit		
Nutritional Components	% of Component/100 g	References
Sugars (g/100 g)	8.92	[12]
vitamin C (mg/100 g)	228.3	
vitamin A (IU/100 g)	624	
Vitamin E (mg/100 g)	0.73	
vitamin K (μg/100 g)	2.6	
Lycopene (in red-fleshed cultivars only); (mg/100 g)	5.2	
Potassium (mg/100 g)	417	
Phosphorus (mg/100 g)	40	
Magnesium (mg/100 g)	22	
Calcium (g/100 g)	18	
**The major nutritional composition of *P. guajava* L. fruit Pulp**		
Protein (g/100 g)	0.3 5.13 ± 0.26 0.88	[13,14,15]
Carbohydrates (g/100 g)	15 13.2	
Vitamin A (IU/100 g)	109 200–400	
Thiamine (B1) (mg/100 g)	0.06 0.046	
Riboflavin (B2) (mg/100 g)	0.06 0.03–0.04	
Niacin (B3) (mg/100 g)	1.3 0.6–1.068	
Ascorbic acid (C) (mg/100 g)	190 100	
Calcium (mg/100 g)	15 9.1–17	
Phosphorus(mg/100 g)	16 17.8–30	
Iron (mg/100 g)	0.3 0.30–0.70	
Potassium (mg/100 g)	292	
Sodium (mg/100 g)	6	
Calories kcal/100 g	54.97 36–50	
**The major nutritional composition of *P. guajava* L. seeds**		
Protein (g/100 g)	11.19 4.8 ± 0.10 7.71	[16,17,18,19,20]
Carbohydrates (g/100 g)	22.2 ± 0.14 11.51	
Vitamin A (IU/100 g)	50.13	
Niacin (B3) (mg/100 g)	0.16	
Ascorbic acid (C) (mg/100 g)	87.44 0.20	
Zinc (mg/100 g)	3.31	
Calcium (mg/100 g)	0.05 ± 0.14 60.07	
Phosphorus(mg/100 g)	160.55	
Iron (mg/100 g)	13.8 3.32	
Potassium (mg/100 g)	300	
Calories kcal/100 g	182	

## 2. Phytochemistry of *P. guajava* L.

### 2.1. LCMS Analysis of the Leaves Extract of P. guajava L.

The literature revealed that UPLC-ESI-QTOF-MS analysis of the leaves extract of *P. guajava* disclosed fourteen compounds (Table 2) of phenolic class of secondary metabolites [21], while in another report, the chemical profiling by HPLC-PDA and LC-TOF-MS (negative and positive modes) showed the presence of altogether 21 compounds (Table 2) from seven *P. guajava* cultivars [22]. Study on pink guava (*P. guajava* L.), the phytochemical investigation through ultra-high performance liquid chromatography with diode array result in identification of 60 phenolics with different structural features, such as flavonoids, ellagitannins, flavones, flavonols, flavanols, proanthocyanidins, dihydrochalcones and anthocyanidins, stilbenes, acetophenones, and benzophenones. Out of all these identified compounds 42 polyphenols were reported for the first time in both peel and flesh, and 24 compounds were detected for the first time in *P. guajava* [23]. However, various chemical profiling of different cultivars of guava have shown the presence of phenolic compounds in major amounts. The dominant phenolic metabolites were assumed to be responsible for its strong antioxidant and antidiabetic activity.

### 2.2. GCMS Analysis of the Leaf Extracts of P. guajava L.

GCMS analysis of the ethanolic extract of guava leaves results in identification of 33 phytochemicals (Table 3). Further studies of ethanolic and aqueous extracts showed the presence of tannin, saponin, polyphenol, flavonoids, steroids, carbohydrate, terpenoids, triterpenoids and glycoside in both the extracts. The quantitative analysis showed phenolics as major constituents (9.33 mg/gm powder), followed by flavonoids (6.42 mg/gm powder), tannin (4.30 mg/gm powder) and saponin (3.67 mg/gm powder) and [24]. Other investigators reported nine compounds through GCMS analysis, which are presented in Table 3. The estimation of total phenolic showed that polyphenolic is present in high amount as in methanol fraction (261.4 ± 8.5) followed by the ethanol (146.7 ± 2.2), ethyl acetate (99.6 ± 2.4), acetone (84.2 ± 2.4), benzene (43.8 ± 2.3) and petrol ether (41.2 ± 1.9) fractions [25]. Ashraf et. al., in 2016 has published the chemical composition of the different extracts of leaves by GCMS and identified the 33 compounds. Further total phenolic contents (mg GAE/mg of plant extract) of methanol (83.34 ± 0.49), chloroform (71.49 ± 0.48) and hexane (53.24 ± 2.05) were determined. The total flavonoids contents (mg QE/mg of plant extract) of methanol, chloroform and hexane were 53.39 ± 0.89, 32.76 ± 1.15, 21.26 ± 1.49 respectively [26]. Another study conducted in 2018 for chemical profiling of the leaves of the guava showed the presence of total nine compounds as presented in Table 3 [27,28]. The analysis of GCMS studies showed interesting data as except some major components such as α-Copaene, Caryophyllene, Epiglobulol, Ledol, Copaene and γ-Muurolene, the extracts of different region possess the different compounds. The above contradictions in results from various groups may be attributed to agro-climatic conditions of the regions, type of cultivar, maturity stage at which the plant was collected, type of extraction technique and polarity of different solvents used.

### 2.3. Chemical Composition of Essential Oil of P. guajava L.

During literature search, several reports were encountered highlighting essential oil compositions of guava fruit and leaves (Table 4). For example, the essential oil obtained through hydrodistillation from the leaves of *P. guajava*, collected from Kathmandu, Nepal, was analyzed by GC-MS. Out of 100 identified compounds 53 were major constituents and accounts for 100% of oil composition. The major phytochemicals identified from essential oil were (*E*)-nerolidol (35.6%) and (*E*)-caryophyllene (15.8%), (2*Z*,6*E*)-farnesol (6.7%), ledol (5.5%) and cubenol (3.99%) [32]. Another report describes leaf essential oil composition of *P. guajava* through GC-MS where 27 substances were identified with α-terpinyl acetate (23.57%), *trans* caryophyllene (17.65%), nerolidol (12.16%), αcadinol (6.71%), α-copaene (6.5%), α humulene (3.92%), (-)-caryphyllene oxide (3.66%), iso aroma-dendren epoxid (2.55%) and *trans* α-bisabolene (2.01%) as the major components. In this report, the overall % age yields of oils were obtained as 0.51% (v/w) [33]. GC-FID and GC-MS analyses of leaf essential oil identified even higher number of components i.e., 46 with major components have been reported as limonene (29.1%), (*E*)-caryophyllene (15.7%), caryophyllene oxide (8.8%), caryophylla-4(12),8(13)-dien-5-ol (6.5%), (*E*)-nerolidol (4.0%), *α*-cadinol (3.4%), muurola-4,10(14)-dien-1-*β*-ol (2.5%), 1,8-cineole (2.6%), *α*-copaene (2.3%) and *α*-humulene (2.0%) [34]., whereas, in other report it is observed that the examined essential oil from leaves of *P. guajava*, dominated by limonene, (*E*)-caryophyllene and (*E*)-nerolidol and their derivatives (Table 4). Since these studies were performed under different conditions and plants used were growing in different climatic conditions, it is reasonable to find varying compositions of essential oil. These variations (Table 4) correspond to climate, type of plant culture and stage at which this fruit was collected.

## 3. Bioactive Compounds Isolated from *P. guajava* L. and Their Structure Activity Relationship

### 3.1. Phenolics/Phenolic Acids from P. guajava L.

**Figure 1:**
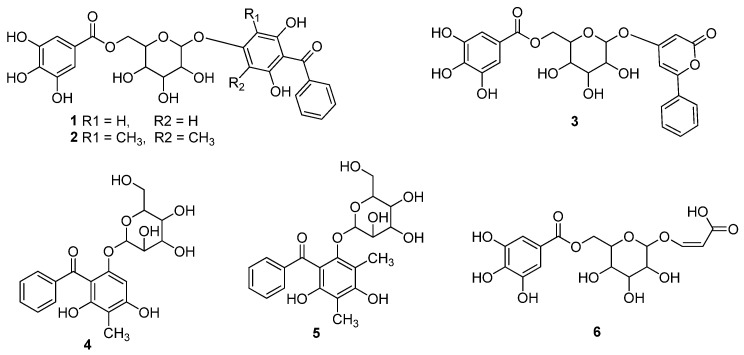
Isolate from the leaves extract of the *P. guajava* L.

The published literature revealed that different research groups have studied secondary metabolic profiles of guava through LCMS/UPLCMS and other related techniques, and have reported phenolics as the major components of guava. Further reports on isolation and structure elucidation of these secondary metabolites have substantiated the above analytical results, since variety of phenolics have been isolated from various parts of this plant. For example, six guavinosides A–F (**1–6**) were separated from the leaf extract of the *P. guajava* (Figure 1), which were identified due to spectroscopic means. Compounds **3** and **6** showed good cytotoxicity against HeLa, SGC-7901 and A549 cell lines, with IC_50_ values of 4.277, 7.288 and 3.246 µg/mL respectively (Table 5). Comparison with potential of reference drug (Adriamycin, IC_50_ = 1.359, 3.118 and 2.684 µg/mL) indicated significant anticancer property of these compounds. In variety of antioxidant assays, compound **3** inhibited the FRAP, DPPH and ABTS activity On the other hand, compound **1** showed a dose dependent FRAP, DPPH and ABTS activity with IC_50_ values of 11.54 (12.5 µg/mL), 14.00 (25 µg/mL), 23.73 (50 µg/mL) and 46.37 (100 µg/mL), followed by significant inhibition by compound **4**, while other compounds also displayed varying potential (Table 5) [36].

**Figure 2:**
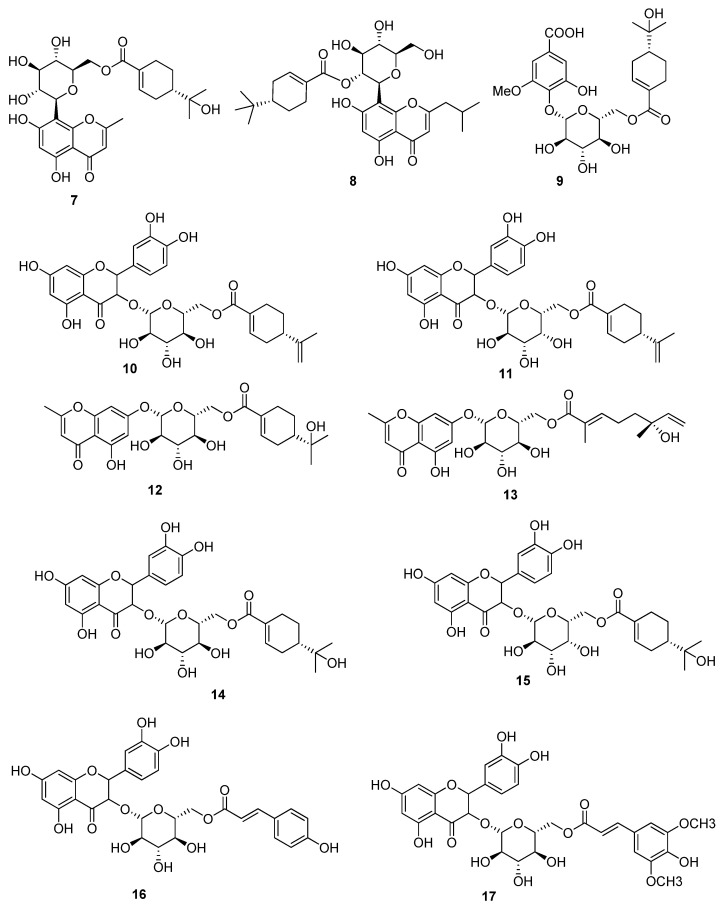
Acylated Phenolic Glycosides (**7**–**17**) were isolated from the leaves of the *P. guajava* L.

**Figure 3:**
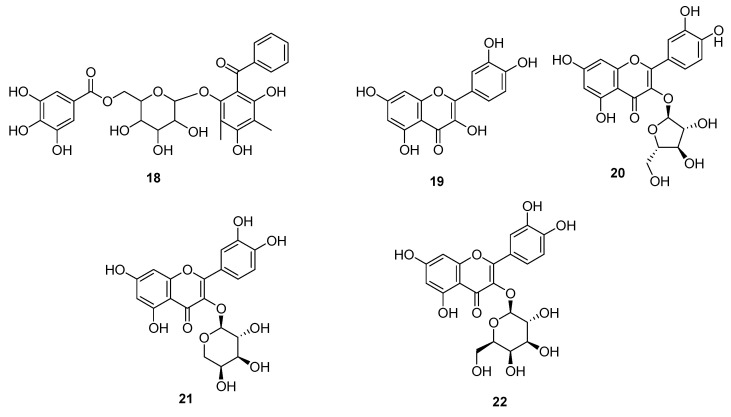
Benzophenone galloyl glycoside (**18**) and flavonoids (**19–22**) were isolated from the leaves of the *P. guajava* L.

Acylated Phenolic Glycosides (**7–17**) were isolated from the leaves of the *P. guajava* (Figure 2), which showed DPPH Scavenged Free Radicals activity with IC_50_ values in the range of 84.28 to 180.00 μM. Since the standard drug ascorbic acid exhibited IC_50_ value of 108.60 μM, it offers a strong candidature of compounds **7–17** to be further studied for their value as antioxidant drug [37]. Compounds **2**, **5** and a benzophenone galloyl glycoside (**18**), were isolated from the leaves of the *P. guajava* L. (Figure 3) by High-Speed Counter-Current Chromatography and their structure was identified through details spectroscopic data. Compound **2**, **5** and **18** were tested against HCT116 and HT29 cells. Compound **2** and **18** showed good inhibitory activity against HCT116 and HT29 cells in a dose dependent and time-dependent manner up to 81.4% at dose of 100 μM after 72 h of treatment, while under same conditions, compound **2** showed less inhibition by 66.2% (Table 5). Furthermore, compound **18** was tested for cellular apoptosis of HCT116 cells and showed good increased in the size of the apoptotic cell population by 1.50-fold (3.65%), 2.33-fold (5.67%), and 10.08-fold (24.53%) at concentration of 40, 60 and 80 μM respectively. It is very important that to mention here that chemical structure of the compound **2** and **18** was nearly similar to that of **5**, and thus their inhibitory potential was compared, which indicated that a trihydroxybenzoate moiety (as in **2** and **18**) is required for anticancer activity of these compounds [38]. Four flavonoids (**19–22**) were also isolated from leaves of the *P. guajava*, and were evaluated for their anticancer and antioxidant activity. Compound **19** showed the FRAP activity on dose dependent manner by a value of 333.26 ± 1.76 (12.5 µg/mL), 359.18 ± 15.14 (25 µg/mL), 379.40 ± 10.31 (50 µg/mL), 401.27 ± 12.23 (100 µg/mL), Compound **20** showed the inhibition with a value of 123.88 ± 14.95 (12.5 µg/mL), 269.00 ± 7.28 (25 µg/mL), 291.63 ± 32.79 (50 µg/mL), 324.58 ± 10.64 (100 µg/mL), Compound **21** displayed the inhibition with a value of 57.21 ± 4.94 (12.5 µg/mL), 175.59 ± 7.11(25 µg/mL), 220.51 ± 22.18 (50 µg/mL), 346.45 ± 25.61 (100 µg/mL) and Compound **22** showed the inhibition with a value of 68.06 ± 5.74 (12.5 µg/mL), 155.89 ± 17.90(25 µg/mL), 287.94 ± 2.26 (50 µg/mL) and 329.68 ± 17.72(100 µg/mL), while these compounds also showed good antioxidant activities on DPPH and ABTS free radicals. The compounds **19–22** displayed the DPPH free radical activity with IC_50_ values of 11.00 ± 0.26, 5 16.13 ± 0.32, 6 13.43 ± 0.12 and 27.03 ± 1.70, respectively and ABTS free radical activity with IC_50_ values of 4.40 ± 0.26, 6.90 ± 0.36, 6.70 ± 0.26, and 10.57 ± 0.51, respectively [36].

### 3.2. Meroterpenoid

**Figure 4:**
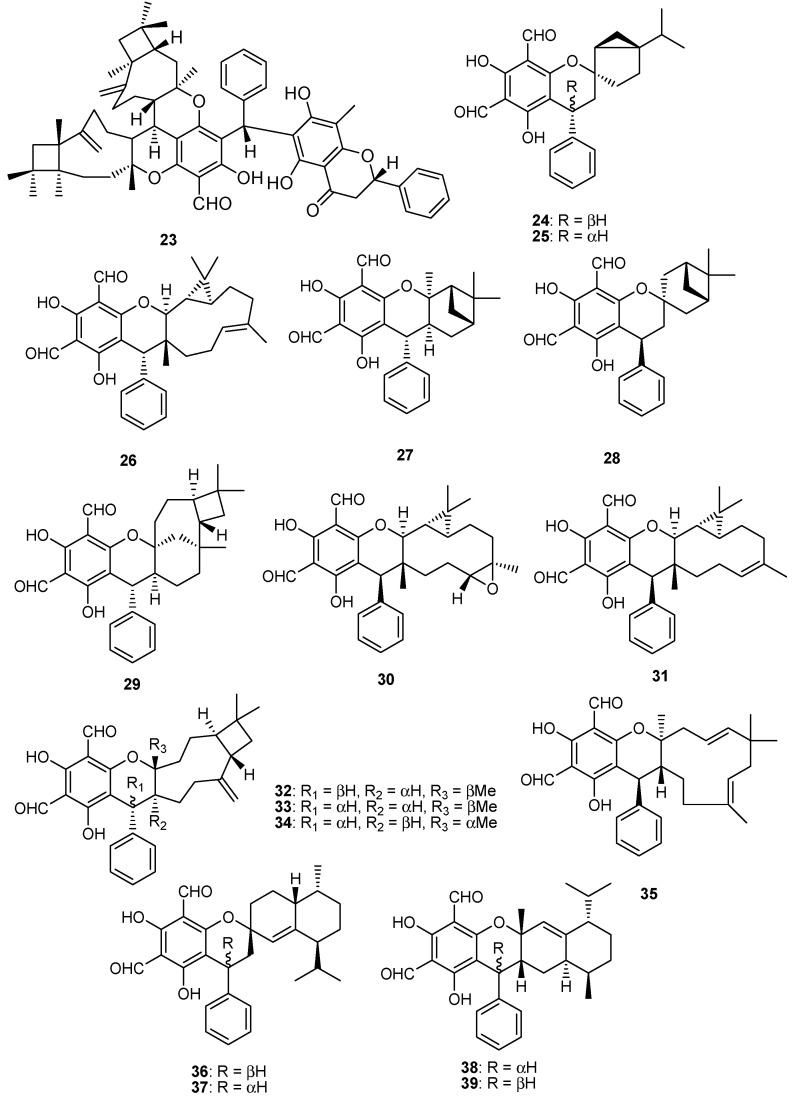
Meroterpenoids isolated from *P. guajava* L.

**Figure 5:**
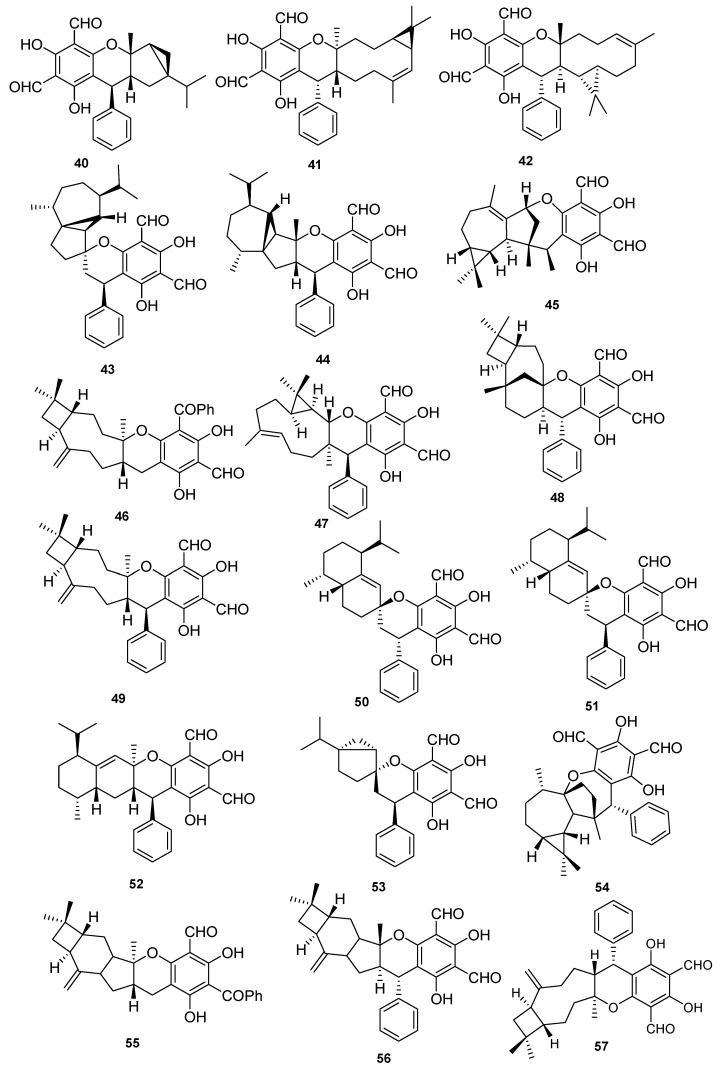
Meroterpenoids isolated from *P. guajava* L.

In addition to above phenolics mostly isolated from leaves of *P. guajava* L., hybrid compounds are noticed as a striking feature of this plant. Among these hybrid compounds, meroterpenoids (Table 4, Table 5, Table 6, Table 7 and Table 8) are the most attractive group of secondary metabolites, in which the terpenoidal part is bonded with other classes of secondary metabolites, especially phenolics, for example, guajavadimer A (**23**), a dimeric sesquiterpene-based meroterpenoid isolated from the leaves of *P. guajava* L. the chemical structure of the compound was established through detail spectroscopic studies and X-ray crystallography. It possessed two caryophyllenes, a flavonone-fused and a benzylphlorogulcinol complicated skeleton and showed moderate hepatoprotective activity against N-acetyl-p-aminophenol (APAP)- induced toxicity in HepG2 cells with value of OD (mean ± SD) 1.654 ± 0.094 [39]. Diformylphloroglucinol-derived meroterpenoids psiguajavadials A (**24**) and B (**25**), guadial A–C (**26–28**), psiguadial B–D (**29–31**), guajadial (**32**), psidial A (**33**), 4,5-diepipsidial A (**34**), guajadial B (**35**) and guajadials C–F (**36–39**) were isolated from *P. guajava* L. and their structure was identified through spectroscopic analysis and ECD calculations [40]. All compounds (**24–39**) showed antitumor activity (Table 5) against HCT116, CCRF-CEM, DU145, Huh7, and A549 with the cell viability rates less than 50% at 60 μM concentration. Compounds **34** and **35** were the most active against A549 cells with IC_50_ values of 160 and 150 μM, respectively. While compounds **24**, **36**, **37** and **39** have same potential at 50 μM, compounds **24**, **25**, **35** and **39** showed dose-dependent inhibition of Top1 activity. Mechanistic study for compounds **24** and **25** displayed potent antiproliferative effects against HCT-116 cells by inducing apoptosis in a dose-dependent manner and induced antagonized Top1-mediated DNA break [41]. Guajadial (**32)**, inhibited endothelial cell proliferation and migration, and furthermore it suppresses tumor growth in human NSCLC (A549 and H1650 cells) xenograft mouse models. This potential has been reported as significant antineoplasmic activity of **32**. Western blotting method to study the underlying mechanisms of VEGF receptor (VEGFR)2-mediated revealed that compound **32** inhibited A549 (IC_50_ = 3.58 μM) proliferation via blocking the Ras/MAPK pathway [42]. Moreover, guadial A–C (**26–28**) [43,44], psiguadial B–D (**22–24**) [41,43], guajadial (**32**) [45], psidial A (**33**) [46], 4,5-diepipsidial A (**34**) [47], guajadial B (**35**) [48] and guajadials C–F (**36–39**) [49] were also previously reported from *P. guajava* L. Among other anticancer meroterpenoids the Diformylphloroglucinol-based guajavadials A–C (**40–42**) were isolated from *P. guajava* L. and the structures of all compounds were determined through spectroscopic studies. All compounds displayed good cytotoxicity against HL-60, A-549, SMMC-7721, MCF-7, and SW480 cancer cell lines with IC_50_ values between 2.28–3.38 µM (Table 5). Compound **40** exhibited the highest potential with a value of IC_50_ = 3.54 µM against SMMC-7721 cell lines, and this activity level is higher than the control drug cisplatin (IC_50_ = 19.82 µM) [50]. The structures activity relationship showed that the arrangement of the isoprene units is responsible for the activity, and thus the terpenoidal skeleton plays key role in activity potential, as can be seen in compounds **41** and **42**. Guajavadial A (**40**) is a 3,5-diformylbenzyl phloroglucinol-coupled monoterpenoid skeleton and guajavadials B (**41**) and C (**42**) are the adducts of the 3,5-diformylbenzyl phloroglucinol and a sesquiterpene with different coupling models [50]. More meroterpenoids namely psiguajadials A–K (**43–53**) along with psiguadial A (**54**), guapsidial A (**55**), and psiguajadial L (**56**) were also isolated from *P. guajava*. All these natural products (**43–56**) has shown significant inhibition of PDE4D-4 with IC_50_ values ranging from 1.34–7.26 μM (Table 6) [51]. This activity potential is comparable with the rolipram, a standard PDE4 inhibitor (IC_50_ 0.62 μM). Since a little difference has been reported in activity level of all these compounds, which may lead to the conclusion that diformylphloroglucinol moiety is required for PDE4D2 inhibitory activities (Table 6). Compounds psiguadial A (**54**) [41], guapsidial A (**55**) [44], and psiguajadial L (**56**) [47] were previously also reported from *P. guajava* produced complex and diverse meroterpenoids bearing phloroglucinol-coupled to sesquiterpenoids or monoterpenes. Similarly, here the Phloroglucinol-coupled to cubebane sesquiterpenoid core in compounds **43** and **44**, and compound **45** has globulane as terpene unit, **48** has caryolane, **49** has caryophyllane, and compounds **50–52** have cadinane unit as terpene unit [51]. One more meroterpenoid Guavadial (**57**) was isolated of *P. guajava* which has caryophyllene combined to diformyl phloroglucinol core [49]. Furthermore, meroterpenoids were obtained from *P. guajava* L. namely Psiguajdianone (**58**), Psiguajanone A (**59**), Psiguajanone B (**60**), Psiguajanone C (**61**), Psiguajanone D (**62**), Psiguajanol A (**63**) along with the already reported Guapsidial A (**55**) and Psiguajadial D (**44**). All compounds were tested for anticancer and anti-inflammatory activity on NO, TNF-α and PEG2 production in RAW264.7 cells. All isolates showed the inhibition with a value ranging from 2.86–11.82, 1.66–31.59 and 1.08–13.63 for NO. TNF-α and PEG2 respectively [52] (Figure 4 and Figure 5).

**Figure 6:**
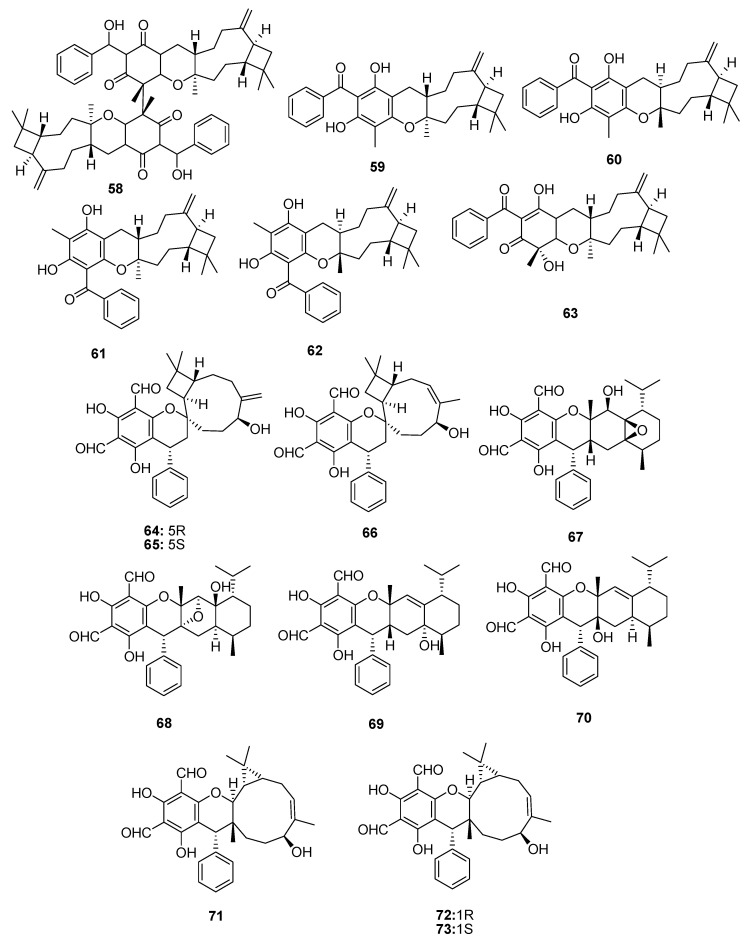
Meroterpenoids isolated from *P. guajava* L.

**Figure 7:**
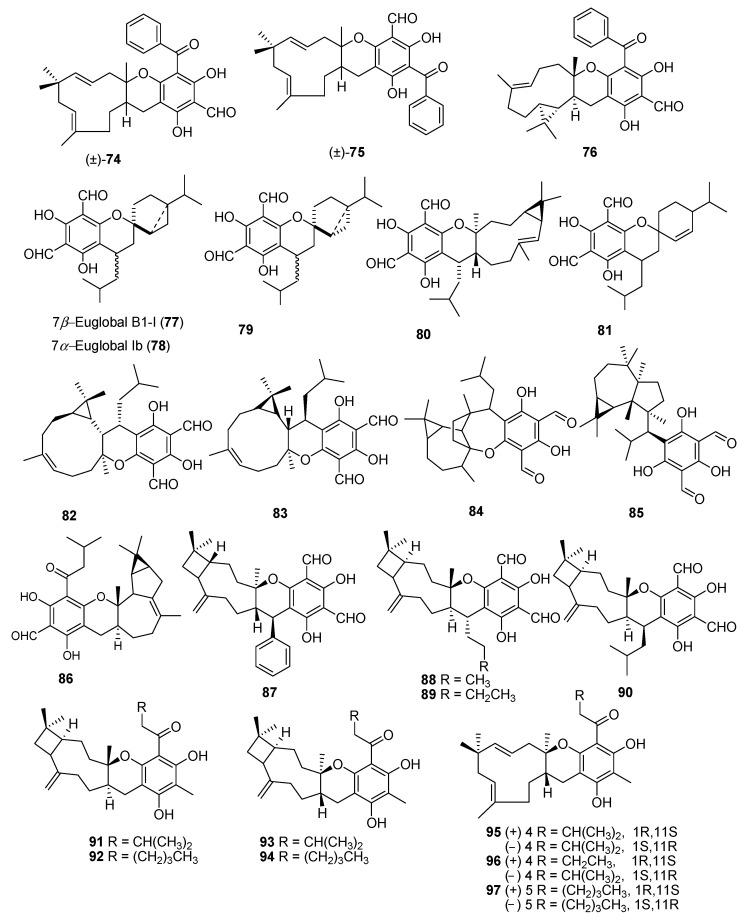
Meroterpenoids isolated from *P. guajava* L.

**Figure 8:**
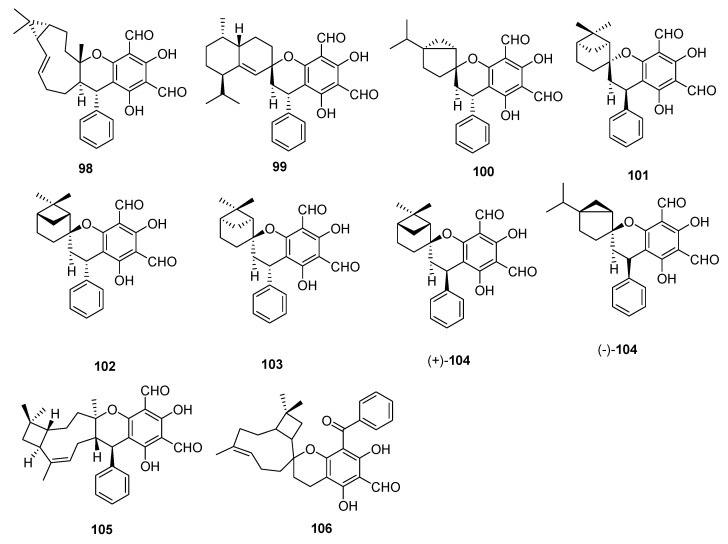
Meroterpenoids isolated from *P. guajava* L.

Psiguadiols A–J (**64–73**), meroterpenoids were isolated from leaves of the *P. guajava* L., which has 6,8-diformyl-5,7-dihydroxy-4-phenylchromane-coupled sesquiterpenoids along with a C-8-spiro-fused 6/6/9/4 tetracyclic ring system. The chemical structure of the natural product was determined from spectroscopic data, ECD data, and single-crystal X-ray diffraction data. The protein tyrosine phosphatase 1B (PTP1B) is critically involved in insulin receptor signaling and an important target from treatment of DM type II. Interestingly guava meroterpenoids, psiguadiols A, G, H (**64**, **70** & **71**) inhibited PTP1B with IC_50_ values of 4.7, 6.2 and 9.2 μM respectively [53], while all other compounds were found inactive. Three psiguamers A–C (**74**–**76**) sesquiterpene-based meroterpenoids bearing a rare methylated benzoylphloroglucinol and bicyclogermacrene units, were isolated from leaves of *P. guajava* L. Their structures were established through comprehensive analysis of spectroscopic data, electronic circular dichroism (ECD) and X-ray crystallographic data. All compounds were tested for anticancer activity but only compound (+)-**74** showed strong cytotoxic activities against five human tumor cell lines; HCT-116, HepG2, BGC-823, A549, and U251 with values of IC_50_; 2.94, 9.01, 6.45, 5.42 and 5.33 μmol/L respectively [54]. Seventeen meroterpenoids were isolated from the leaves of the *Psidium guajava* including euglobal B1-1 (**77**), euglobal Ib (**78**), euglobal Ic (**79**), euglobal III (**80**), euglobal IIb (**81**), euglobal-Iva (**82**), euglobal Ivb (**83**), euglobal V (**84**), macrocarpal A (**85**), ecalrobusone E (**86**), guajadial C (**36**), guajadial D (**37**), guajadial E (**38**), guajudial (**87**), psiguajadial H (**50**), psiguajadial I (**51**), and psiguajadial J (**52**) were isolated from the leaves of guava [37]. Jejuguajavones A–J (**88–97**) isolated from the 95% EtOH extract of Jejuguava leaves and evaluated against the PTP1B enzyme. Compound **88–91** showed the good inhibitory activity with value ranging from 9.40–37.83 µM [55]. Eleven polycyclic phloroglucinol meroterpenoids guajamers A–I (**98–106**), along with guadial A (**26**) guadial C (**28**) were isolated from the leaves of *P. guajava*. All compounds were checked for antibacterial activity and **99**–**104**, **26** and **28** showed moderate activity [56]. It is concluded from the bioactivity of the isolated compounds that *P. guajava* L. is excellent sources for the treatment of oxidative stress, diabetes and inflammation. Further compounds isolated from *P. guajava* L. leaves and their anti-cancer effects against human cancer cells, paving the way for these compounds and *P. guajava* L. leaves to be used as possible chemoprevention agents against cancer (Figure 6, Figure 7 and Figure 8).

## 4. Pharmacological Activities

### 4.1. Antidiabetic Potential of Leaf Extract

Literature search revealed that several studies have been carried out to evaluate pharmacological potential of guava leaves. The guava leaves have an ability to increase glycogen synthesis and halt the process of hepatic gluconeogenesis by regulating AMPK/ACC pathway in streptozotocin-induced diabetic rats, when orally administered as 200 mg/kg by weight [57]. It is further reported that guava leaves reduced triglycerides, phospholipids, free fatty acids, total cholesterol and LDL levels while HDL level was raised in STZ induced rats [57]. To validate the role of guava leaves in treatment of Diabetes Mellitus (DM), a novel purified heteropolysaccharide GP70-3M from the leaves was tested in vitro against α-glucosidase, which showed outstanding inhibitory activity with an IC_50_ value of 2.539 µM. This potential has been reported to be 1867 times greater than control acarbose (IC_50_ value of 4.744 mM). Different parts of the plant were screened for their diabetic inhibition (Table 7) [58].

**Table 7:** Anti-diabetic potential of different parts of *P. guajava*.

Activity	Enzyme	IC_50_ μM	Plant Part Used	References
Anti-diabetic	α-glucosidase	2.539	Leaves	[58]
	α-glucosidase	1.0	Leaves	[59]
	α-glucosidase	0.5	Bark	[59]
	α-amylase	10.6	Bark	[59]
	Glucose-6-Phosphatase	Significant	Leaves	[58]
Control	Acarbose	4.744		[58]

**Table 8:** IC_50_ and selectivity index (SI) of essential oil of *P. guajava* leaves (PG-EO) against diferent cell lines.

Cell Line	Treatment (µg/mL)			
	PG-EO		DXR	
	IC50	SI	IC50	SI
GM07492A	126.4 ± 11.8	-	0.5 ± 0.2	-
MCF-7	96.9 ± 8.4 ^a^	1.3	62.1 ± 2.0	-
HeLa	128.7 ± 1.5	-	5.3 ± 1.3	-
M059J	103.6 ± 5.1 ^a^	1.2	16.2 ± 2.5	-

The leaf extract of *P. guajava* increased the uptake of 2-deoxy-d-[1–3H]-glucose in C_2_C_12_ muscle cells with a value of 161.4 ± 10.1%, *p* = 0.0015 as compared to vehicle control (dimethyl sulfoxide), and standard drugs metformin (144.0 ± 7.7%, *p* = 0.0345) and insulin (141.5 ± 13.8%, *p* = 0.0495). Furthermore, it also noted that leaf extract of *P. guajava* considerably enhanced the triglyceride accumulation in 3T3-L1 cells compared to standard drug (rosiglitazone) [59].

### 4.2. Anticonvulsant Effects

The *P. guajava* leaf ethanolic extract has been found to afford anti-convulsant activity; since it exerted dose dependent (200 mg/kg and 400 mg/kg) effect on seizures induced mice using MES as suggested by some parameters for example reduced hind-limb tonic extension (HLTE), increased percentage protection from induced MES convulsions. Anti-convulsant effect on Maximal Electrocshock (MES) mice model suggests that the extract has produced dose dependent anticonvulsant effect in albino mice. While in pentylenetetrazole (PTZ) induced mice model *P. guajava* extract prolonged the clonic convulsion latency, reduced convulsion duration in a dose dependent manner along with reduction in seizure score [60].

### 4.3. Antiproliferative Potential of Essential Oils from P. guajava Leaves Extracts

Essential oil obtained from leaves of *P. guajava* was evaluated for its antiproliferative activity against human gliobastoma (M059J), human cervical adenocarcinoma (HeLa), breast adenocarcinoma (MCF-7) and normal human GM07492A cell lines, whereas lung fibroblasts cell line was used as control. The reported results showed that the oil exhibited significant IC_50_ values for M059J and MCF-7 compared to normal line as presented in Table 8 [61].

### 4.4. Antimutagenic Effect

The methanolic extract of *P. guajava* leaves tested against methyl sulfonate (MMS), sodium azide (NaN_3_), benzo(a)pyrene (BP) and 2-aminofluorene for its antimutagenic effect. The methanol extract of *P. guajava* leaf extract was found to inhibit 70% of mutagenesis at concentration of 80 mg/mL. This setup is on the basis that the phenolic contents present in *P. guajava* have broad-spectrum antimutagenic activity and could serve as potentially good *Candidates* for phytomedicine [25].

### 4.5. Antifungal Properties

Various published work revealed that *P. guajava* L. has also shown to possess some antifungal activities. Its tannins and flavonoid fraction were analyzed for antifungal activity using 21 different compounds in fraction of two, having phenolic compound in higher concentration. In an in vitro assay performed against three fungal strains of *Candida* i.e *albicans*, *krusei* and *tropicalis* through microdilution showed that IC_50_ values for these strains ranged from 69.29 to 3444.62 μg/mL for the isolated and combined fractions of flavonoids and tannins. While the reference compound fluconazole and combined fraction ranged from 1.57 to 925.56 μg/mL, which shows that natural products have some synergism with antifungal potential. The fractions were found to affect pleomorphism capacity as well along with inhibition of fungal strain in isolated form, enhancing the action potential of Fluconazole, reducing the concentration and hindering the morphological transition, one of the main virulence factor of *Candida* genus [62]. The *P. guajava* leaves extract was used for microbiological assays to determine IC_50_, inhibitory effect of associated fraction with Fluconazole against *Candida* species and cell viability curve through microdilution method. Antifungal bioassay performed on solid media by modifying morphological and fungicidal concentration and results ranged between 5.10 and 926.56 mg/mL revealed the effect of change in concentration effects the inhibition [21]. This suggests that *P. brownianum* and *P. guajava* can potentially be used to develop drugs to treat fungal infections [63,64].

### 4.6. Antiparasitic Potential

*P. guajava* L. and *Psidium browninaum* Mart ex DC leaf aqueous and hydroethanolic extracts were tested for their antiparasitic and cytotoxic potential against *Leishmania braziliensis*, *Trypanosoma cruzi* epimastigote forms, *L. infantum* promastigotes and fibroblasts at three different concentrations (250, 500 and 1000 μg/mL). The *T. cruzi* forms were not inhibited by the extracts from *P. guajava* L. *P. guajava* showed small amount of activity against both *L. braziliensis* and *L. infantum*. As for cytotoxicity aqueous decoction extract of *P. browninaum* showed highest percentage among all other extracts and showed mortality rate of 90.85% for fibroblast at 1000 μg/mL [65].

### 4.7. Anthelmintic Activity

The aqueous extract of *P. guajava* paralyzes the nematodes both Levamisole-sensitive and Levamisole-resistant strains of *Caenorhabditis elegans*, in a dose dependent manner. Different studies were carried out by applying concentration-dependent doses. At a concentration of 25 mg/mL of the *P. guajava* extract, 100% paralysis of the wild type worms was achieved with in 4 h. A similar effect was observed for N2 wild type and CB193 resistant worms and egg-laying ability was decreased by 40% at the same concentration. These reports disclose that *P. guajava* extracts have also potential anthelminic effect against nematodes [66]. Further these studies revealed the presence of triterpene responsible for anthelmintic activity. Therefore, it is also concluded that secondary metabolites from *P. guajava* leaves extract could serve as basis for antileishmanial drugs [67].

### 4.8. Antioxidant Potential

Phytochemistry of Guava showed the presence of flavonoids and phenolics, which is in agreement with antioxidant activity. The antioxidant activity for crude extract of peels, flesh and seed was 264.30 ± 5.39 μmol TE/g dw, 98.78 ± 3,40 μmol TE/g dw and 62.84 ± 2.81 μmol TE/g dw, respectively, which is nearly equal to the genotype known as Fan Retief and Advanced Selection. The crude extract of the peel part having highest antioxidant activity has a greater proportion of antioxidant compounds [68]. The products in *P. guajava* leaf tea (GLT) contain both phenolic forms i.e., soluble and insoluble-bound. The fermentation process via *Saccharomyces cerevisiae* and *Monascus* anka followed by hydrolysis through complex enzymes increases the soluble phenolic form. Free radical scavenging (DPPH) results of guava were found in close agreement with standard Trlox and ascorbic acid. The excellent IC_50_ in μM revealed the guava extract as potential anti-oxidant. Comparative studies on extracts and Trolox revealed the direct relationship with concentration. As the concentration was increased, the reducing ability was also enhanced and, like radical scavenging activity, SPFE reducing power was greater than rest of the extracts. The reducing power for SPFE = 97.86 mmol TE/g DM, SPF = 50.3 mmol TE/g DM, SPUF = 7.4 mmol TE/g DM. The reducing ability of soluble phenolics (SPF/SPFE) was higher than insoluble bound phenolics (IBF/IBFE) at same concentration. However, the reducing power for insoluble-bound phenolics of unfermented GLT (IBUF) was found to be 11.4 mmol TE/g DM which greater than IBF (2.9 mmol TE/g DM) and IBFE (3.5 mmol TE/g DM). When the extracts were tested for their inhibitory activity against α-glucosidase, the inhibitory effect was promisingly high for SPUF, but it was observed that it was much higher for fermented extract. The IC_50_ values for inhibition of α-glucosidase were in order SPFE (IC 50 = 11.8 µg/mL) > SPF (IC 50 = 19.2 µg/mL) > SPUF (IC 50 = 29.1 µg/mL). Moreover, insoluble-bound phenolics of fermented GLT (IBPF) and IBPFE have low inhibitory effect on α-glucosidase (i.e., IC_50_ = 104.4 µg/mL and IC _50_ = 112.2 µg/mL respectively) as compared to insoluble-bound phenolics of unfermented GLT (IBPUF) having IC_50_ = 71.6 µg/mL. The IC_50_ value for positive control (acarbose) was significantly greater than all the extracts i.e., IC_50_ = 178.52 µg/mL [69].

### 4.9. Suppression of Osteoarthritis

The leaves extract from *P. guajava* and ellagic acid, a polyphenolic compound from the extract, have role in degeneration of aggrecan at the onset of osteoarthritis (OA) by halting activity of metalloproteinase and disintegrin with throbospondin-type-5. The efficacy of extract along with ellagic acid was determined on destruction of cartilage by giving extract as a constituent of diet of anterior cruciate ligament-transected rats (ACLT). The results suggested that *P. guajava* leaves extract have role in suppressing progression of OA in ACLT rats and inhibition of joint destruction at early stages through ellagic acid-mediation [70].

### 4.10. Antidiarrheal Activity

The antidiarrheal effect of *P. guajava* leaf extract carried out at normal rats and diarrheal rats suggested promising effect. Four groups were formed using normal rats: low-dose *P. guajava* leaves extract, high dose *P. guajava* leaves extract, control and gallic acid while 5 groups were formed using diarrheal rats: low-dose *P. guajava* leaves extract, high-dose *P. guajava* leaves extract, desmopressin, untreated control group and gallic acid. The low-dose *P. guajava* extract was equal to 50 mg/kg, high-dose *P. guajava* extract was equal to 100 mg/kg for both normal and diarrheal rats while desmopressin was used 0.2 mg/kg for a period of one month. The administration of *P. guajava* leaves extract to diarrheal rats stabilized all the parameters such as kidney weight decline, levels of potassium, sodium and chloride in serum, urine volume, serum urea etc. along with antidiarrheal effect *P. guajava* leaves extracts also aids in protein conservation [71]. According to survey, there is no single clinical trial found on intake of guava as active anti-diarrheal ingredient.

### 4.11. Antiestrogenic Activity

The Guajadial, meroterpenoids, from *P. guajava* leaves extract reported to have antiproliferative and antiestrogenic activity, the action mechanism is similar to tamoxifen which indicates it as promising therapeutic agent based on phytoestrogen. The enriched fraction of guajadial form crude *P. guajava* leaves extract has selectivity and antiproliferative activity in vitro against human breast cancer cell lines MCF-7 and MCF-7 BUS. The total growth inhibition for MCF-7 was 5.59 µg/mL and for MCF-7 BUS was 2.27 µg/mL. The in vivo analysis on uterus of pre-pubescent rats also confirmed the antiestrogenic activity as guajadial fraction halted the proliferative activity of estradiol [72].

### 4.12. Anticancer Potential

#### 4.12.1. Anticancer Activity of Leaves Extract

The ethanolic extract of *P. guajava* leaves and quercetin isolated fractions reduce CCl_4_-induced cytotoxic effect on HepG2 cell lines. The levels of GSH, viability and cytotoxicity were reduced in CCl_4_ treated cell lines while lipid peroxidation, Lactate dehydrogenase (LDH), Alanine aminotransferase (ALT) and Aspartate aminotransferase (AST) were increased. The levels of all these parameters were regulated in a positive manner through the application of *P. guajava* leaves extract [73].

#### 4.12.2. Cytotoxic Effect of *P. guajava* Fruit Extract

With the useful biological effects, it is necessary to determine the cytotoxicity of any drug, formulation and nutraceutical. The *P. guajava* extract administered orally at 2000 mg/kg and 5000 mg/kg b.w of mice did not make any noticeable change in number of kidney podocyte, liver hepatocyte and body weight. The extract from *P. guajava* leaves extract is safe to use as it non-toxic to both kidney and liver [74]. The aqueous extract of *P. guajava* leaves in the diet of *Oreochromis niloticus* not only increased body weight but also increased the villi surface area by increasing its length and width. The immune response and antioxidant activity were improved as total protein content (glutathione S-transferase, superoxide dismutase, and glutathione peroxidase) increased. The *Aeromonas hydrophila*, a pathogen towards *O. niloticus*, inhibited by the presence of *P. guajava* leaves extract, while the absence of extract in the diet of fish increased the mortality rate [75]. In 2019 Babatola et al. also evaluated the toxicity of aqueous leaves extract of three different species of guava i.e., pink, red and white. The rats were used as test animals for this study and extract was administered at a dose of 50, 500 and 5000 mg/kg bodyweight for a period of 14 days. Observation of experimental periods showed effect on parameters accounted and found no toxic effects with a slight increase in body weight but no deaths. The estimated LD_50_ was found to be 50–5000 mg/kg for these pink, red and white leaves extract of guava [76].

### 4.13. Antiviral Activity

The antiviral selectivity of *P. guajava* leaves extract was determined against herpes simplex virus 1 and human immunodeficiency virus, and median cytotoxicity and half-maximal effective concentration were obtained. The EC_50_ values for HIV-1 strains and HSV-1 were ranged between 0.05 and 3 mg/mL and below 0.2 mg/mL respectively. Antiviral activity of guava extract was found to be based on flavonoid and phenolic contents as HPLC analysis results revealed the presence of phenols (0.8 to 2.1 GAE mg/mL) and flavonoids (62.7 to 182.1 Rutin Eq mg/g DW) [77]. Some direct studies on *P. guajava* isolated compounds quercetin, catechin, and gallic acid have antiviral activity against Dengue virus. The catechin is best among the three of them as it showed 100% inhibition (pre-treatment) and 91.8% inhibition (post-treatment) depending upon the experimental strategies [78].

### 4.14. Antibacterial Activity

In 2019, a study conducted by E. A. J. Silva and his colleagues evaluated antibacterial activity for essential oil present in *P. guajava* leaves. The essential oil has shown moderate activity for various genus of *Streptococcus*. The activity was described in terms of MIC values *S. sobrinus* has MIC value of 100 µg/mL, *S. mitis* has MIC value of 200 µg/mL, *S. mutans* has MIC value of 200 µg/mL), *S. salivarius* has MIC value of 200 µg/mL and *sanguinis* has MIC value of 400 µg/mL [61]. The crude extracts obtained from *P. guajava* leaves were evaluated for antimicrobial activities by Priscilla Alexander et al. 2019 have evaluated the antimicrobial activities. Crude extractions have mixed fraction of saponins, flavonoids, tannins, glycosides, terpenoids and steroids. The antimicrobial screening showed that crude extracts were strongly active against *S. faecalis*, *E. coli* and *S. aureus* having MIC value of 5.00 mg/mL. In contrast, ethanolic extracts were more actively involved in inhibition and mean zone values were found out to be 6.72 ± 0.01 for *S. faecalis* and 10.44 ± 0.02 for *E. coli* [79]. *P. guajava* have also been tested for its antibacterial activity as a toothpaste. Three different formulations (F) were made F1, F2 and F3 having leaves powder of *P. guajava* 10, 15 and 20 mg respectively. Therefore, results revealed that the antimicrobial potential against *Streptococcus mutants*, *Streptococcus oralis, Proteus vulgaris*, *Bacillus subtilis* and *Staphylococcus aureus* strains concentration dependent and F3 formulation found best one. Among all bacterial strains, the best inhibition found for *Proteus vulgaris* i.e., 1.1 cm while lowest zone inhibition was found against *Staphylococcus aureus* i.e., 0.5 cm [28]. Therefore, Patel et al. 2019 investigate an important factor regarding the medium of extraction and he evaluated the water extract of *P. guajava* leaves as anti-infective against *Staphylococcus aureus* and *Pseudomonas aeruginosa* present in *Caenorhabditis elegans* nematode host. The extracts were prepared through three different methods i.e., Decoction, Vacuum Assisted Extraction (VAE) and Microwave Assisted Extraction (MAE). The proved that extracts prepared through MAE showed better activity while its anti-infective activity was than compared to hydroalcoholic extract against five pathogenic bacteria, which were obtained using same extraction technique. Both the extracts had capability to reduce the virulence of all strains (*Serratia marcescens*, *S. aureus*, *Chromobacterium violaceum*, and *P. aeruginosa*) except *S. pyrogens* towards *C. elegans* (Table 9). This lead to reveal the method of extraction effects the activity. According to them, it seems that anti-infective property of these extracts is somehow related to property of quorum modulation that can modulate production of pigments related to quorum sensing within these susceptible bacteria [80,81].

### 4.15. Acute and Sub-Acute Toxicity of P. guajava Leaves Extract

The acute and subacute toxicity level of *P. guajava* bark extract was evaluated using Wistar rats. The extract was proved non-toxic and non-lethal and estimated LD_50_ was found to be >5000 mg/kg body weight for acute toxicity. The variations in relative weight of organs, body weight and other biochemical parameters that were significant were taken into account in treated animals and control group. Single dose administration at 5000 mg/kg body weight is non-toxic while repeated administration at 1000 mg/kg body weight produced sex-specific toxic effect i.e., minor liver inflammation was observed in females. Hence Psidium plant proved to have mild organ toxicity but have hepatoprotective and hematological potency [82].

### 4.16. Antimicrobial Activity of Essential Oils of P. guajava Leaves

The essential oils present in leaves of *P. guajava* have known to have cytotoxic and antimicrobial activities. Their antimicrobial activity was determined against three Gram-positive (*Streptococcus aureus*, *Enterococcus faecalis* and *Staphylococcus aureus*) and three Gram-negative strains (Escherichia coli, *Pseudomonas aeruginosa* and Haemophilus influenzae). The antimicrobial activity of oil was significant against both Gram-positive and Gram-negative strains and ranges between 0–13 mm while no cytotoxicity was observed using brine shrimp lethality bioassay [83]. The essential oil reportedly inhibit two bacterial human pathogens with MIC values that range from 0.065–0.261 mg/mL while it also inhibits some pathogenic fungi in plants i.e., 82.80% inhibition of *Fusarium chlamydosporum* and 86.02% inhibition of *Curvularia lunata* [34].

### 4.17. P. guajava Leaves Activity against Diarrhea

An antidiarrheal activity of *P. guajava* leaves was clinically measured and three different doses (6-leaf, 10-leaf, and 14-leaf) of *P. guajava* leaves decoction extracts were used to their ability against diarrhea. The 14-leaf (7.4 g) decoction proved to be the most successful in the testing. Patients who received the decoction three times per day were able to return to normalcy in 72 h as opposed to 120 h for controls. Haemoglobin, liver, and kidney indicators were all within normal limits, which demonstrated the intervention’s safety [84].

## 5. Industrial Applications of *P. guajava*

Dyes and pigments are used in numerous industries worldwide, although the discharge of these materials presents significant risks to the natural environment. Nowadays, water contamination is one of the main causes of environmental pollution. Different synthetic dyes are released directly into natural water resources that are potentially pollutant the resources and make it unfit for domestic and agricultural use. On the other hand, the aromatic structures of these dyes give them greater stability and their degradation process is very slow. Further their oxidation through different oxidizing agents is not easy. Thus, these materials become main pollutants to environments. So, there is a crucial need to find environmentally friendly and cost-effective materials and methods to remove these materials from environment. *P. guajava* L. leaves nanocomposites materials were widely studies for decontamination of these pollutants. In a recent study a silver: iron oxide (α-Fe_2_O_3_-Ag) nanocomposite was prepared for decontamination of chromium (VI) ions from water. Further it is observed that the Cr(VI) adsorption on Fe_2_O_3_-Ag surface is endothermic and spontaneous in nature. The adsorbed Cr(VI) can easily be recovered (α-Fe_2_O_3_-Ag) nanocomposite and used up to five times [85]. In another study *P. guajava* leaves were used as biosorbents for the removal of Brilliant Green (BG) [86]. Magnetic nanohybrid composite γ-Fe_2_O_3_@GL was prepared by incorporated the Maghemite nanoparticles into framework of *P. guajava* leaves. γ-Fe_2_O_3_@GL was developed for water purification and found efficient for adsorption of methylene blue [87].

Plant-derived proteases are widely used in food and pharmaceutical industries. The upward requirement for biologic-based enzymes, in the food and pharmaceutical industries, has made them an interesting topic for physiologists and biochemists. The existence of two pH optima of *P. guajava* leaves protease suggests that at least two major proteases are present in it [21]. An environmentally friendly and cost-effective material CuONPs was biosynthesized by using *P. guajava* L. leaf. It showed potential antibacterial activity against Gram-positive and Gram-negative bacteria. It is non-toxic and exhibited good photocatalytic degradation for Congo red (CR) and methylene blue (MB). The SnO_2_ nanoparticles within the size of 8 to 8 nm were synthesized by using *P. guajava* L. leaves extract. These nanoparticles photocatalytic activity was analyzed and found effective for photo degradation of reactive yellow 186. A novel, eco-friendly cotton gauze fabric was synthesized by using *P. guajava* leaves powder extract. The outer membrane of Biocompatible microcapsules was synthesized from *P. guajava* leaves powder extract, starch core and calcium-alginate (Ca-alginate). This product was found effective for medical uses. Another novel, eco-friendly and cost-effective material, tungsten oxide nanorods (WO_3_ NRs), was synthesized by using *P. guajava* leaves extract. These nanorods were found prodigious in photocatalytic degradation of reactive green 19 (RG 19) dye.

## 6. Conclusions and Future Prospects

The results demonstrated that almost all parts of *P. guajava* are rich in diverse secondary metabolites, especially phenolics, flavonoids, squalene and vitamin E. This feature makes the plant a potential source of antioxidants to be used in nutraceuticals and functional food products. Essential oil analysis of this plant indicated the presence of caryopyllene and a variety of its derivatives, which makes *P. guajava* an anti-inflammatory agent. Striking feature of *P. guajava* is that all its parts are rich in meroterpenoids specially derived from phloroglucinol, which are mainly produced by different fungi with immunosupressive activity. Leaf and bark extracts can be used as a natural source of α-glucosidase inhibitors. In addition, the bark extract of *P. guajava* was an effective α-amylase inhibitor. Moreover, *P. guajava* leaf extract improved glucose uptake in muscle cells, while both leaf and bark extracts enhanced the triglyceride content in adipocytes in culture. *P. guajava* leaf and bark extracts may thus hypothetically have future applications in the treatment of type 2 diabetes. Similar to this, meroterpenoids’ isolation and activity against many cancer cell lines make it a crucial source for the development of anticancer drugs. The domestic applications of leaves also indicate its important in the field of medicine. Additionally, its applications in industry for the development of numerous beneficial products makes it a significant source that demands special consideration from the scientific community. Overall, it can be said that *P. guajava* is a useful plant and a rich supplier of nutrients for human growth.

## Figures and Tables

**Table 2:** Phytochemicals identified through LCMS studies of the Fresh Leaf of *P. guajava* (A).

Sr. No.	Rt (min)	Compound	References
**UPLC-ESI-QTOF-MS analysis of the leaves extract of *P. guajava***			
1	0.98	HHDP glucose Isomer	[21]
2	1.865	Gallic acid	
3	2.421	Galocatechin	
4	3.197	Catechin	
5	4.08	Quercetin-galloylhexoside Isomer	
6	4.292	Ellagic acid	
7	4.665	Reynoutrin	
8	5.557	Guavinoside B	
9	6.889	9(*S*),12(*S*),13(*S*)-Trihydroxy-10 (*E*),15(*Z*)-octadecadienoic acid	
10	9.839	Naringenin	
11	2.430	Gallocatechin	
12	2.877	Procyanidin B Isomer	
13	4.387	Quercetin	
14	6.005	2,6-dihydroxy-3-methyl-4-*O*-(600-*O*-galloyl-b-d- glucopyranosyl)benzophenone	
**Phytochemicals identified through HPLC-PDA and LC-TOF-MS (negative and positive modes) in the *P. guajava* cultivars.**			
15	14.3	Delphinidin 3-*O*-glucoside	[22]
16	15.1	Cyanidin-3-*O*-glucoside	
17	16.0	Myricetin-3-*O*-b-d-glucoside	
18	17.1	Myricetin-3-*O*-arabinoside	
19	18.3	Myricetin-3-*O*-xyloside	
20	19.0	Quercetin-3-*O*-galactoside	
21	19.5	Quercetin-3-*O*-glucoside	
22	20.0	Quercetin-3-*O*-a- arabinoside	
23	20.6	Avicularin	
24	21.0	Isorhamnetin-3-*O*-glucoside	
25	21.9	Isorhamnetin-3-*O*-galactoside	
26	24.0	Abscisic acid (co-injection)	
27	26.1	Quercetin (co-injection)	
28	26.4	Pinfaensin	
29	27.8	Gallocatechin-(4a-8)gallocatechol	
30	28.6	Turpinionosides A	
31	29.1	Pedunculoside	
32	30.6	Gallocatechin-(4a-8)catechin	
33	35.6	Guavenoic acid	
34	38.5	Madecassic acid	
35	45.2	Asiatic acid	
**Compounds identified in peel and flesh of ripe pink guava (*P. guajava* L. cv. ‘Criolla’).**			
**Phenolic acid derivatives**			
36	1.80	Galloyl-hexoside	[23]
37	2.10	Galloyl-hexoside	
38	3.01	Gallic acid^d^	
39	5.70	Galloyl-pentoside	
40	7.40	Hydroxybenzoyl-galloylglucoside	
41	11.25	Dimethoxycinnamoyl-hexoside	
42	11.30	Dimethoxycinnamoyl-hexoside	
**Flavones**			
43	11.95	Chrysin-*C*-hexoside	
**Ellagitannins**			
44	13.75	Valoneic acid bilactone	
**Flavonols**			
45	12.16	Quercetin-galloyl-hexoside	
46	12.30	Quercetin-hexoside	
47	12.50	Quercetin-hexoside	
48	12.90	Quercetin-glucuronide	
49	13.39	Quercetin-pentoside	
50	13.60	Quercetin-pentoside	
51	13.92	Quercetin-pentoside	
52	16.67	Quercetin-galloyl-pentoside (guavinoside C)	
53	17.95	Quercetin-deoxyhexoside-hexoside	
54	18.63	Quercetin	
**Monomeric Flavanols**			
55	4.50	Gallocatechin	
56	6.01	Epigallocatechin	
57	16.55	Catechin	
58	7.81	Epicatechin	
59	9.50	Gallocatechin gallate	
60	10.53	Epigallocatechin gallate	
61	11.63	Catechin gallate	
62	13.47	Epicatechin gallate	
**Proanthocyanidins**			
63	2.71	PAC B-Type (*E*)GCg-(*E*)GC	
64	3.81	PAC B-Type (*E*)GC-(*E*)GC	
65	4.64	PAC B-Type (*E*)GC-(*E*)GC	
66	4.93	PAC B-Type (*E*)GC-(*E*)C	
67	5.03	PAC B-Type (*E*)GC-(*E*)C	
68	5.48	PAC B-Type (*E*)C-(*E*)GC	
69	5.97	PAC B-Type (*E*)GC-(*E*)C	
70	6.79	PAC B-Type (*E*)GC-(*E*)GC	
71	6.80	PAC B-Type (*E*)C-(*E*)C	
72	7.07	PAC B-Type (E)C-(*E*)GC-(*E*)GC	
73	7.26	PAC B-Type (*E*)Cg-(*E*)C	
74	8.32	PAC B-Type (*E*)C-(*E*)GC	
75	8.44	PAC B-Type (*E*)GC-(*E*)C	
76	8.67	PAC B-Type (*E*)C-(*E*)C-(*E*)C	
77	10.28	PAC B-Type (*E*)C-(*E*)C	
78	13.10	PAC B-Type (*E*)Cg-(*E*)GC	
79	14.35	PAC B-Type (*E*)Cg-(*E*)GC	
80	20.07	PAC B-Type (*E*)C-(*E*)GC	
**Dihydrochalcones**			
81	12.00	Phloretin-C-glucoside (nothofagin)	

**Table 3:** GCMS analysis of the leaves extracts of *Psidium guajava*.

Peak No.	R. Time	Area %	Name	References
**GCMS analysis of the ethanolic extract of guava leaves**				
**1**	3.582	0.37	Butanoic acid, 2-methyl-, methyl este	[29]
**2**	8.436	0.57	dl-Limonene $$ Cyclohexene, 1-me	
**3**	8.534	1.37	1,8-Cineole $$ 2-Oxabicyclo [2.2.2]	
**4**	11.783	0.20	(*E*)-2,6-Dimethyl-5,7-octadien-2-ol	
**5**	14.013	0.34	Cyclohexasiloxane, dodecamethyl-	
**6**	15.555	0.22	Alpha.-Copaene	
**7**	16.776	19.76	Trans-Caryophyllene	
**8**	17.793	2.44	Alpha.-Humulene	
**9**	18.438	1.12	Germacrene d	
**10**	19.115	7.48	Trans-alpha.-bisabolene	
**11**	19.233	0.44	Aromadendrene 2 $$	
**12**	19.351	9.75	Beta.-Bisabolene	
**13**	19.949	1.19	Delta.-Cadinene	
**14**	20.124	0.36	(-)-Endo-2,6-dimethyl-6-(4-methyl-	
**15**	20.381	1.09	CIS-.alpha.-bisabolene $$	
**16**	20.923	21.87	Nerolidol B (CIS OR TRANS	
**17**	21.803	10.55	(-)-Caryophyllene oxide	
**18**	22.174	0.35	Trans-Caryophyllene	
**19**	22.444	0.59	Humulene oxide $$	
**20**	22.807	0.69	Germacrene d	
**21**	22.917	0.68	Tricyclo [3.3.1.13,7]decane, 2-brom	
**22**	23.099	3.29	(+)-Aromadendrene	
**23**	23.202	4.93	Torreyol $$ 1-Naphthalenol	
**24**	23.441	1.91	Globulol $$ (-)-Globulol	
**25**	23.631	1.46	Beta.-Bisabolol	
**26**	23.963	1.36	Alpha.-bisabolol	
**27**	25.711	0.20	2-Methyl-6-(trimethylsilyl)benzophe	
**28**	26.512	0.92	8-Acetyl-3,3-epoxymethano-6,6,7-t	
**29**	27.223	0.35	1,2-Benzenedicarboxylic acid, dibut	
**30**	28.671	1.89	1,2-Benzenedicarboxylic acid, buty	
**31**	32.600	0.71	Propionic acid, 2-isopropo	
**32**	38.268	0.82	Bis(2-ethylhexyl) phthalate	
**33**	48.521	0.72	Hexadeca-2,6,10,14-tetraen	
**Components of *P. guajava* methanol fraction as identified by GC-MS analysis.**				
**34**	3.43	10.48	2-Nonanone	[25]
**35**	3.49	1.30	1-Heptanamine	
**36**	13.84	3.10	7-Oxabicyclo [4.1.0] heptanes, 3-oxir	
**37**	15.19	5.13	2-Diethylamino-4-phenylthiooct-2-e	
**38**	15.27	14.29	4-Methylthiazole	
**39**	18.89	4.42	2-Propenoic acid, 3-phenyl-, (*E*)-	
**40**	19.23	11.62	13-Tetradecenal	
**41**	19.44	7.10	2-Butyne, 1,4-dichloro	
**42**	21.32	2.60	1,2-Benzenedicarboxylic acid, 3-ni	
**Chemical components of methanol, chloroform and hexane extracts of *P. guajava* analyzed by gas chromatography mass spectroscopy (GC-MS).**				
**43**	5.19	-	Hydroxydimethylacetic acid	[26]
**44**	13.88	-	Tridecyl trifluoroacetate	
**45**	14.47	-	*n*-Cetane	
**46**	15.59	-	Farnesan	
**47**	16.10	-	Pyrogallol	
**48**	16.42	-	α-Copaene	
**49**	17.21	-	Caryophyllene	
**50**	17.35	-	Aromandendrene	
**51**	17.83	-	Dodecyliodide	
**52**	17.83	-	Heneicosane	
**53**	17.89	-	Alloarmadendrene	
**54**	18.04	-	γ-Muurolene	
**55**	18.08	-	Eicosane	
**56**	18.09	-	Tetracosane	
**57**	18.41	-	β-Bisabolene	
**58**	18.58	-	β-Chamigrene	
**59**	19.11	-	α-Calacorene	
**60**	19.50	-	Cetene	
**61**	19.83	-	Caryophyllene oxide	
**62**	19.83	-	α-Bulnesene	
**63**	20.13	-	Epiglobulol	
**64**	20.14	-	Ledol	
**65**	20.40	-	cis-Thujopsene	
**66**	20.69	-	Copaene	
**67**	21.15	-	Culmorin	
**68**	23.13	-	E-15-Heptadecenal	
**69**	23.48	-	cis-Z--Bisabolene epoxide	
**70**	27.27	-	Palmitic acid	
**71**	31.15	-	Stearic acid	
**72**	32.55	-	Hexachlorodisiloxane	
**73**	40.22	-	Squalene	
**74**	44.12	-	Vitamin E	
**75**	47.63	-	β-Sitosterol	
**Identified compounds from the ethyl acetate extract of *P. guajava* leaves**				
**76**	9.601	-	2-Isopropoxyethylamine	[30]
**77**	14.468	-	Bicyclo(7.2.0) undec-4-Ene,4,11,11-Trimethyl-8methylene-,[IR-(IR*,4Z,9S*)]-	
**78**	16.374	-	Caryophyllene	
**79**	16.754	-	Alpha-Farnesene	
**80**	16.874	-	Trans-Z,alpha-Bisaboleneepoxide	
**81**	17.804	-	Alpha-bisabolol	
**82**	19.850	-	Β-carotene	
**83**	22.116	-	Propanoic acid, 2-(Aminooxy)-	
**84**	25.948	-	2,4,6-Cycloheptatrien-1-one,3,5-Bis-Trimethylsilyl-	
**Identification of phytochemicals in guava leaf extract by GCMS**				
**85**	6:22.5	-	Caryophyllene	[31]
**86**	5:55.80		α-Copaene	
**87**	6:34.10		*Cis*-muurola-3,5-diene	
**88**	6:35.80		Humulene	
**89**	6:44.60		Cyclosativene	
**90**	6:50.30		Bicyclo [5.3.0]decane, 2-methylene-5-(1methylvinyl)-8-methyl-	
**91**	6:54.70		*cis*-à-Bisabolene	
**92**	7:00.20		1*H*-Benzocycloheptene, 2,4a,5,6,7,8,9,9a-octahydro-3,5,5-trimethyl9-methylene-, (4aS-*cis*)-	
**93**	7:08.90		1*H*-Cyclopropa[a] naphthalene, 1a,2,3,5,6,7,7a,7b-octahydro-1,1,7,7a-tetramethyl-, [1aR-(1aà,7à,7aà,7bà)]	
**94**	7:09.20		Naphthalene, 1,2,3,5,6,8a-hexahydro4,7-dimethyl-1-(1-methylethyl)-, (1S-*cis*)	
**95**	7:12.80		Benzene, (1,3,3-trimethylnonyl)-	
**96**	7:24.90		Cadala-1(10),3,8-triene	
**97**	7:26.90		1,6,10-Dodecatrien-3-ol, 3,7,11-trimethyl-, (E)-	
**98**	7:48.10		Spathulenol	
**99**	7:46.60		Cubenol	
**100**	7:57.40		6S-2,3,8,8-Tetramethyltricyclo [5.2.2.0(1,6)]undec2-ene	
**101**	8:02.80		2-Hydroxy-2,4,4-trimethyl3-(3-methylbuta-1,3-dienyl)cyclohexanone	
**102**	8:04.40		Torreyol	
**103**	8:08.20		α-Cadinol	
**104**	8:09.20		5,6,6-Trimethyl-5-(3-oxobut1-enyl)-1-oxaspiro [2.5] octan-4-one	
**105**	8:13.30		α-Bisabolol	
**106**	9:28.10		Tetradecanoic acid, trimethylsilyl ester	
**107**	9:28.30		10-Undecynoic acid, trimethylsilyl ester	
**108**	11:00.30		Hexadecanoic acid, trimethylsilyl ester	
**109**	24:54.3		Isoamyllaurate	

**Table 4:** Phytochemicals identified from the leaf essential oil extracted from *P. guajava*.

Sr. No.	Compound	RT (min)	Area %	References
**Essential oil composition from leaf of** **P. guajava** **from Kathmandu, Nepal.**				
1	Limonene	1028	0.17	[32]
2	1,8-Cineole	1031	tr	
3	Benzyl alcohol	1032	0.07	
4	Linalool	1100	0.27	
5	*trans-p*-Mentha-2,8-dien-1-ol	1120	tr	
6	Chrysanthenone	1124	tr	
7	*cis-p*-Mentha-2,8-dien-1-ol	1134	tr	
8	*trans-p*-Menth-2-en-1-ol	1139	tr	
9	Terpinen-4-ol	1176	0.11	
10	(3*Z*)-Hexenyl butanoate	1187	tr	
11	α-Terpineol	1189	0.90	
12	Nerol	1226	0.64	
13	(3*Z*)-Hexenyl 2-methylbutanoate	1231	tr	
14	Geraniol	1252	1.48	
15	Methyl geranate	1324	tr	
16	Benzyl butanoate	1346	tr	
17	α-Cubebene	1349	0.14	
18	Neryl acetate	1365	0.17	
19	α-Copaene	1375	2.82	
20	(3*Z*)-Hexenyl hexanoate	1382	tr	
21	Geranyl acetate	1385	0.50	
22	β-Cubebene	1390	tr	
23	(*E*)-Caryophyllene	1419	15.80	
24	*cis*-Muurola-3,5-diene	1451	0.22	
25	α-Humulene	1453	2.53	
26	*allo*-Aromadendrene	1460	0.46	
27	Dauca-5,8-diene	1474	0.18	
28	α-Amorphene	1477	0.33	
29	Germacrene D	1481	tr	
30	*trans*-Muurola-4(14),5-diene	1492	0.21	
31	*epi*-Cubebol	1495	1.01	
32	α-Muurolene	1501	0.30	
33	(*Z*)-α-Bisabolene	1504	0.35	
34	β-Bisabolene	1510	0.34	
35	Cubebol	1516	1.30	
36	δ-Cadinene	1525	4.03	
37	*trans*-Cadina-1,4-diene	1533	1.44	
38	(*E*)-Nerolidol	1569	35.59	
39	(3*Z*)-Hexenyl benzoate	1573	0.28	
40	Caryophyllene oxide	1584	2.59	
41	Gleenol	1587	0.19	
42	Ledol	1601	5.51	
43	1,10-di-*epi*-Cubenol	1616	0.27	
44	1-*epi*-Cubenol	1628	2.42	
45	Caryophylla-4(12),8(13)-dien-5-ol	1636	0.41	
46	Cubenol	1643	3.99	
47	α-Muurolol	1646	3.05	
48	β-Eudesmol	1651	0.34	
49	α-Cadinol	1655	1.91	
50	*epi*-β-Bisabolol	1671	0.39	
51	α-Bisabolol	1686	0.12	
52	Shyobunol	1690	0.46	
53	(2*Z*,6*E*)-Farnesol	1723	6.70	
**Chemical Composition of Leaf Essential Oil of *P. guajava* L. from North East India**				
54	α-Pinene	5.48	0.33	[33]
55	Benzaldehyde	6.16	0.33	
56	α-Myrcene	6.32	0.53	
57	α-Terpinyl acetate	7.18	23.57	
58	p-Metha-*trans*-2,8-dien-1-ol	8.81	0.09	
59	Citronella	8.94	0.09	
60	*cis*-p-Menthe-1(7),8-dien-2-ol	9.61	0.24	
61	3-Cyclohexene-1-methanol, α, α-4-trimethyl-	9.74	0.99	
62	Nerol	10.05	0.28	
63	*trans*-p-Metha-1(7),8-dien-2-ol	10.24	0.32	
64	α-Copaene	12.49	6.50	
65	*trans*-Caryophyllene	13.55	17.65	
66	α-Humulene	14.20	3.92	
67	γ-Muurolene	14.48	0.73	
68	*trans*-α-bisabolene	14.90	2.01	
69	(+)-e-Cadinene	15.44	3.18	
70	*cis*-Calamenene	15.60	1.97	
71	Naphthalene	15.80	1.92	
72	Nerolidol	16.40	12.16	
73	Caryophyllene oxide	17.09	3.66	
74	Ledol	17.55	1.98	
75	Epiglobulol	17.67	1.40	
76	*iso*-Aromadendrene epoxide	17.96	2.55	
77	α-Cadinol	18.33	6.71	
78	Viridiflorol	18.63	2.38	
79	α-Cedrene	19.17	0.38	
80	Ledeneoxide-(II)	20.14	0.03	
**Phytochemicals indentifed from the leaf essential oil extracted from *P. guajava* by GC-FID and GC-MS from Lucknow India**				
81	*α*-Pinene	-	0.8	[35]
82	Benzaldehyde	-	0.6	
83	*β*-Pinene	-	0.2	
84	6-Methyl-5-hepten-2-one	-	t	
85	Myrcene	-	0.4	
86	*α*-Phellandrene	-	0.1	
87	*p*-Cymene	-	0.2	
88	Limonene	-	29.1	
89	1,8-Cineole	-	2.6	
90	(*Z*)-*β*-Ocimene	-	t	
91	(*E*)-*β*-Ocimene	-	0.1	
92	*γ*-Terpinene	-	0.1	
93	Terpinolene	-	t	
94	Linalool	-	t	
95	Terpinen-4-ol	-	0.1	
96	*α*-Terpineol	-	0.5	
97	*cis*-Carveol	-	0.4	
98	Neryl acetate	-	0.1	
99	*α*-Copaene	-	2.3	
100	(*E*)-Caryophyllene	-	15.7	
101	*α*-Humulene	-	2.0	
102	*allo*-Aromadendrene	-	0.3	
103	*γ*-Muurolene	-	0.6	
104	Germacrene D	-	0.1	
105	Viridiflorene	-	1.2	
106	*α*-Selinene	-	1.3	
107	*α*-Muurolene	-	t	
108	(*Z)*-*α*-Bisabolene	-	t	
109	*β*-Bisabolene	-	0.3	
110	*γ*-Cadinene	-	0.2	
111	*δ*-Cadinene	-	1.7	
112	*trans*-Cadina-1,4-diene	-	0.5	
113	*α*-Calacorene	-	0.1	
114	(*E*)-Nerolidol	-	4.0	
115	Caryophyllenyl alcohol	-	0.3	
116	Caryophyllene oxide	-	8.8	
117	Gleenol	-	0.1	
118	Ledol	-	0.2	
119	Humulene epoxide II	-	1.3	
120	1-*epi*-Cubenol	-	0.2	
121	Muurola-4,10(14)-dien-1-β-ol	-	2.5	
122	Caryophylla-4(12),8(13)-dien-5-ol	-	6.5	
123	*epi*-*α*-Muurolol	-	t	
124	*α*-Muurolol	-	1.1	
125	*α*-Cadinol	-	3.4	
126	(2*Z*,6*E*)-Farnesol	-	0.9	

**Table 5:** Antioxidant and anticancer activities of isolated compounds from *P. guajava* L.

Test Compounds	Antioxidant	Anticancer	References
Guavinoside A (**1**)	DPPH = IC_50_ 37.93 ± 0.50 µg/mL, ABTS = IC_50_ 13.63 ± 1.25 µg/mL, FRAP = IC_50_ (12.5 µg/mL) 18.46 ± 7.50, IC_50_ (25 µg/mL) 53.29 ± 16.84, IC_50_ (50 µg/mL) 131.03 ± 21.31, IC_50_ (100 µg/mL) 174.60 ± 16.36	SGC = >10, A549 = > 10, Hela = > 10	[36]
Guavinoside B (**2**)	DPPH = IC_50_ 8.30 ± 1.35 µg/mL, ABTS = IC_50_ 5.47 ± 0.65 µg/mL, FRAP = IC_50_ (12.5 µg/mL) 48.30 ± 10.71, IC_50_ (25 µg/mL) 90.63 ± 8.10, IC_50_ (50 µg/mL) 185.03 ± 19.38, IC_50_ (100 µg/mL) 341.94 ± 12.90	SGC = >10, A549 = > 10, Hela = > 10	
Guavinoside C (**3**)	DPPH = IC_50_ 13.07 ± 0.57 µg/mL, ABTS = IC_50_ 5.47 ± 0.65 µg/mL, FRAP = IC_50_ (12.5 µg/mL) 91.28 ± 12.92, IC_50_ (25 µg/mL) 143.76 ± 28.44, IC_50_ (50 µg/mL) 250.53 ± 24.02, IC_50_ (100 µg/mL) 386.38 ± 25.31	SGC = IC_50_ 4.277 µg/mL, A549 = IC_50_ 7.288 µg/mL, Hela = IC_50_ 3.246 µg/mL	
Guavinoside D (**4**)	DPPH = IC_50_ > 100 µg/mL, ABTS = IC_50_ 29.27 ± 0.67 µg/mL, FRAP = IC_50_ (12.5 µg/mL) ND, IC_50_ (25 µg/mL) ND, IC_50_ (50 µg/mL) ND, IC_50_ (100 µg/mL) ND	SGC = > 10, A549 = > 10, Hela = > 10	
Guavinoside E (**5**)	DPPH = IC_50_ >100 µg/mL, ABTS = IC_50_ 32.97 ± 3.5 µg/mL, FRAP = IC_50_ (12.5 µg/mL) 11.54 ± 1.33, IC_50_ (25 µg/mL) 14.00 ± 4.43, IC_50_ (50 µg/mL) 23.73 ± 3.73, IC_50_ (100 µg/mL) 46.37 ± 1.27	SGC = > 10, A549 = > 10, Hela = > 10	
Guavinoside F (**6**)	DPPH = IC_50_ 13.10 ± 0.10 µg/mL, ABTS = IC_50_ 5.93 ± 0.21 µg/mL, FRAP = IC_50_ (12.5 µg/mL) 70.64 ± 16.29, IC_50_ (25 µg/mL) 152.02 ± 13.98, IC_50_ (50 µg/mL) 263.31 ± 14.12, IC_50_ (100 µg/mL) 413.94 ± 14.36	SGC = IC_50_ 4.277 µg/mL, A549 = IC_50_ 7.288 µg/mL, Hela = IC_50_ 3.246 µg/mL	
guajanoside A (**7**)	DPPH = IC_50_ 131.87 ± 4.12 μM		[37]
guajanoside B (**8**)	DPPH = IC_50_ 113.33 ± 3.44 μM		
guajanoside C (**9**)	DPPH = IC_50_ 114.94 ± 3.19 μM		
guajanoside D (**10**)	DPPH = IC_50_ 97.68 ± 1.88 μM		
guajanoside E (**11**)	DPPH = IC_50_ 97.05 ± 7.83 μM		
cypellocarpin C (**12**)	DPPH = IC_50_ > 200 μM		
eucamalduside A (**13**)	DPPH = IC_50_ 180.00 ± 3.57μM		
cypellogin A (**14**)	DPPH = IC_50_ 103.95 ± 0.06 μM		
cypellogin B (**15**)	DPPH = IC_50_ 98.75 ± 1.22 μM		
quercetin-3-*O*-β- d-(6″-*O*-p-coumaroyl)-galactopyranoside (**16**)	DPPH = IC_50_ 92.55 ± 4.03μM		
Guavaric A (**17**)	DPPH = IC_50_ 84.28 ± 4.68 μM		
3,5-dihydroxy-2,4- dimethyl-1-*O*-(6′-O-galloyl-β- d -glucopyranosyl)-benzophenone (**18**)		HCT116 = at 40 μM by 1.50-fold (3.65%), at 60 by 2.33-fold (5.67%), at 80 μM by 10.08-fold (24.53%)	[38]
quercetin (**19**)	FRAP = IC_50_ (12.5 µg/mL) 333.26 ± 1.76, IC_50_ (25 µg/mL) 359.18 ± 15.14, IC_50_ (50 µg/mL) 379.40 ± 10.31, IC_50_ (100 µg/mL) 401.27 ± 12.23		[36]
quercetin-3-*O*-a- L -arabino- furanoside (**20**)	FRAP = IC_50_ (12.5 µg/mL) 123.88 ± 14.95, IC_50_ (25 µg/mL) 269.00 ± 7.28, IC_50_ (50 µg/mL) 291.63 ± 32.79, IC_50_ (100 µg/mL) 324.58 ± 10.64		
quercetin-3-*O*-a- l -arabinopyranoside (**21**)	FRAP = IC_50_ (12.5 µg/mL) 57.21 ± 4.94, IC_50_ (25 µg/mL) 175.59 ± 7.11, IC_50_ (50 µg/mL) 220.51 ± 22.18, IC_50_ (100 µg/mL) 346.45 ± 25.61		
querce- tin-3-*O*-b- *D* -galactopyranoside (**22**)	FRAP = IC_50_ (12.5 µg/mL) 68.06 ± 5.74, IC_50_ (25 µg/mL) 155.89 ± 17.90, IC_50_ (50 µg/mL) 287.94 ± 2.26, IC_50_ (100 µg/mL) 329.68 ± 17.72		
Guajavadimer A (**23**)	-	HepG2 = OD (mean ± SD) 1.654 ± 0.094	[39]
Psiguajavadial A (**24**)	-	HCT116 = IC_50_ 7.60 µM, CCRF-CEM = IC_50_ 25.2 µM, DU145 = IC_50_ 20.2 µM, Huh7 = IC_50_ 48.8 µM, A549 = IC_50_ 2.99 µM	[40,41,42,43,44,45,46,47,48,49]
Psiguajavadial B (**25**)		HCT116 = IC_50_ 21.6 µM; CCRF-CEM = IC_50_ 9.63 µM; DU145 = IC_50_ 26.3 µM; Huh7 = IC_50_ 13.7 µM; A549 = IC_50_ 0.90 µM	
Guadial A (**26**)		HCT116 = IC_50_ 5.74 µM; CCRF-CEM = IC_50_ 2.95 µM; DU145 = IC_50_ 5.35 µM; Huh7 = IC_50_ 28.0 µM; A549 = IC_50_ 9.62 µM	
Guadial B (**27**)		HCT116 = IC_50_ 26.5 µM; CCRF-CEM = IC_50_ 6.72 µM; DU145 = IC_50_ 18.0 µM; Huh7 = IC_50_ 55.3 µM; A549 = IC_50_ 13.4 µM	
Guadial C (**28**)		HCT116 = IC_50_ 13.0 µM; CCRF-CEM = IC_50_ 12.9 µM; DU145 = IC_50_ 14.5 µM; Huh7 = IC_50_ 29.6 µM; A549 = IC_50_ 5.70 µM	
Psiguadial B (**29**)		HCT116 = IC_50_ 15.5 µM; CCRF-CEM = IC_50_ 18.2 µM; DU145 = IC_50_ 43.3 µM; Huh7 = IC_50_ 47.0 µM; A549 = IC_50_ 8.73 µM	
Psiguadial C (**30**)		HCT116 = IC_50_ 14.4 µM; CCRF-CEM = IC_50_ 9.3 µM; DU145 = IC_50_ 49.1 µM; Huh7 = IC_50_ 10.8 µM; A549 = IC_50_ 3.06 µM	
Psiguadial D (**31**)		HCT116 = IC_50_ 7.0 µM; CCRF-CEM = IC_50_ 2.59 µM; DU145 = IC_50_ 6.08 µM; Huh7 = IC_50_ 5.20 µM; A549 = IC_50_ 1.07 µM	
Guajadial (**32**)		HCT116 = IC_50_ 20.4 µM; CCRF-CEM = IC_50_ 0.87 µM; DU145 = IC_50_ 11.8 µM; Huh7 = IC_50_ 20.7 µM; A549 = IC50 2.42 µM	
Psidial A (**33**)		HCT116 = IC_50_ 11.5 µM; CCRF-CEM = IC_50_ 17.4 µM; DU145 = IC_50_ 11.9 µM; Huh7 = IC_50_ 47.0 µM; A549 = IC_50_ 14.1 µM	
4,5-diepipsidial A (**34**)		HCT116 = IC_50_ 9.13 µM; CCRF-CEM = IC_50_ 7.0 µM; DU145 = IC_50_ 4.79 µM; Huh7 = IC_50_ 2.82 µM; A549 = IC_50_ 0.16 µM	
Guajadial B (**35**)		HCT116 = IC_50_ 3.54 µM; CCRF-CEM = IC_50_ 7.58 µM; DU145 = IC_50_ 16.4 µM; Huh7 = IC_50_ 2.93 µM; A549 = IC_50_ 0.15 µM	
Guajadial C (**36**)		HCT116 = IC_50_ 4.42 µM; CCRF-CEM = IC_50_ 42.8 µM; DU145 = IC_50_ 55.4 µM; Huh7 = IC_50_ 2.93 µM; A549 = IC_50_ 33.6 µM	
Guajadial D (**37**)		HCT116 = IC_50_ 0.61 µM; CCRF-CEM = IC_50_ 16.0 µM; DU145 = IC_50_ 30.3 µM; Huh7 = IC_50_ 44.09 µM; A549 = IC_50_ 36.2 µM	
Guajadial E (**38**)		HCT116 = IC_50_ 4.69 µM; CCRF-CEM = IC_50_ 12.7 µM; DU145 = IC_50_ 23.2 µM; Huh7 = IC_50_ 51.5 µM; A549 = IC_50_ 18.4 µM	
Guajadial F (**39**)		HCT116 = IC_50_ 3.66 µM; CCRF-CEM = IC_50_ 7.80 µM; DU145 = IC_50_ 27.7 µM; Huh7 = IC_50_ 11.1 µM; A549 = IC_50_ 13.8 µM	
Guajavadial A (**40**)		HL-60 = IC_50_ 4.73 µM; A-549 = IC_50_ 5.62 µM; SMMC-7721 = IC_50_ 4.37 µM; MCF-7 = IC_50_ 22.28 µM; SW480 = IC_50_ 14.55 µM	[50]
Guajavadial B (**41**)		HL-60 = IC_50_ 6.49 µM; A-549 = IC_50_ 5.78 µM; SMMC-7721 = IC_50_ 5.05 µM; MCF-7 = IC_50_ 18.02 µM; SW480 = IC_50_ 13.07 µM	
Guajavadial C (**42**)		HL-60 = IC_50_ 3.38 µM; A-549 = IC_50_ 5.66 µM; SMMC-7721 = IC_50_ 3.54 µM; MCF-7 = IC_50_ 14.54 µM; SW480 = IC_50_ 18.97 µM	
Psiguajadial A (**43**)			[41,44,47,51]
Psiguajadial B (**44**)			
Psiguajadial C (**45**)			
Psiguajadial D (**46**)			
Psiguajadial E (**47**)			
Psiguajadial F (**48**)			
Psiguajadial G (**49**)			
Psiguajadial H (**50**)			
Psiguajadial I (**51**)			
Psiguajadial J (**52**)			
Psiguajadial K (**53**)			
Psiguadial A (**54**)			
Guapsidial A (**55**)		HepG2 = IC_50_ 45.93 ± 6.83 μM, Hela = IC_50_ 39.33 ± 6.21 μM	
Psiguajadial L (**56**)			
Psiguajdianone (**58**)		HepG2 = IC_50_ 27.90 ± 0.85 μM, Hela = IC_50_ 28.14 ± 2.79 μM	[52]
Psiguajanone A (**59**)			
Psiguajanone B (**60**)			
Psiguajanone C (**61**)		HepG2 = IC_50_ 27.45 ± 2.32 μM	
Psiguajanone D (**62**)			
Psiguajanol A (**63**)			
Psiguadiols A (**64**)			[53]
Psiguadiols G (**70**)			
Psiguadiols H (**71**)			
psiguamers A ((+)**74**)		HCT-116 = IC_50_ 2.94 μmol/L, HepG2 = IC_50_ 9.01 μmol/L, BGC-823 = IC_50_ 6.45 μmol/L, A549 = IC_50_ 5.42 μmol/L, and U251 = IC_50_ 5.33 μmol/L	[54]

**Table 6:** Anti-inflammatory and Enzyme inhibitory activities of isolated compounds from *P. guajava* L.

Test Compounds	Anti-Inflammatory	Enzyme Inhibitory	References
Guadial A (**26**)		PDE4D-4 = IC_50_ 2.70 μM	
Guajadial (**32**)		PDE4D-4 = IC_50_ 1.62 μM	
Guajadial C (**36**)		PDE4D-4 = IC_50_ 2.28 μM	
Guajadial D (**37**)		PDE4D-4 = IC_50_ 1.93 μM	
Guajadial E (**38**)		PDE4D-4 = IC_50_ 2.73 μM	
Guajadial F (**39**)		PDE4D-4 = IC_50_ 2.67 μM	
Guajavadial A (**40**)		PDE4D-4 = IC_50_ 2.01 μM	[50]
Psiguajadial A (**43**)		PDE4D-4 = IC_50_ 3.11 μM	[41,44,47,51]
Psiguajadial B (**44**)		PDE4D-4 = IC_50_ 5.03 μM	
Psiguajadial C (**45**)		PDE4D-4 = IC_50_ 4.50 μM	
Psiguajadial D (**46**)	NO = IC_50_ 11.82 ± 1.17 μM, TNF-α = IC_50_ 31.59 ± 3.18 μM, PEG_2_ = IC_50_ 13.63 ± 0.59 μM	PDE4D-4 = IC_50_ 4.14 μM	
Psiguajadial E (**47**)		PDE4D-4 = IC_50_ 3.25 μM	
Psiguajadial F (**48**)		PDE4D-4 = IC_50_ 2.63 μM	
Psiguajadial G (**49**)		PDE4D-4 = IC_50_ 1.34 μM	
Psiguajadial H (**50**)		PDE4D-4 = IC_50_ 1.81 μM	
Psiguajadial I (**51**)		PDE4D-4 = IC_50_ 2.51 μM	
Psiguajadial J (**52**)		PDE4D-4 = IC_50_ 2.53 μM	
Psiguajadial K (**53**)		PDE4D-4 = IC_50_ 3.68 μM	
Psiguadial A (**54**)		PDE4D-4 = IC_50_ 7.26 μM	
Guapsidial A (**55**)	NO = IC_50_ 11.15 ± 0.42 μM, TNF-α = IC_50_ 37.64 ± 3.83 μM, PEG_2_ = IC_50_ 15.38 ± 0.39 μM	PDE4D-4 = IC_50_ 5.61 μM	
Psiguajadial L (**56**)		PDE4D-4 = IC_50_ 1.37 μM	
Psiguajdianone (**58**)	NO = IC_50_ 9.07 ± 0.27 μM, TNF-α = IC_50_ 12.57 ± 0.56 μM, PEG_2_ = IC_50_ 4.27 ± 0.55 μM		[52]
Psiguajanone A (**59**)	NO = IC_50_ 6.11 ± 0.09 μM, TNF-α = IC_50_ 1.88 ± 0.83 μM, PEG_2_ = IC_50_ 6.36 ± 1.27 μM		
Psiguajanone B (**60**)	NO = IC_50_ 5.72 ± 1.19 μM, TNF-α = IC_50_ 2.10 ± 0.72 μM, PEG_2_ = IC_50_ 4.00 ± 0.74 μM		
Psiguajanone C (**61**)	NO = IC_50_ 4.36 ± 0.36 μM, TNF-α = IC_50_ 5.97 ± 1.52 μM, PEG_2_ = IC_50_ 2.24 ± 0.65μM		
Psiguajanone D (**62**)	NO = IC_50_ 7.38 ± 2.60 μM, TNF-α = IC_50_ 1.66 ± 0.26 μM, PEG_2_ = IC_50_2.09 ± 0.06 μM		
Psiguajanol A (**63**)	NO = IC_50_ 2.86 ± 0.95μM, TNF-α = IC_50_ 5.28 ± 1.56 μM, PEG_2_ = IC_50_ 1.08 ± 0.08 μM		
Psiguadiols A (**64**)		PTP1B = IC_50_ 4.7 μM,	[53]
Psiguadiols G (**70**)		PTP1B = IC_50_ 6.2 μM,	
Psiguadiols H (**71**)		PTP1B = IC_50_ 9.2 μM,	
psiguamers A ((+)**74**)			[54]
Jejuguajavones A (**88**)		PTP1B = IC_50_ 10.52 ± 0.71 μM,	[55]
Jejuguajavones B (**89**)		PTP1B = IC_50_ 37.83 ± 3.54 μM,	
Jejuguajavones C (**90**)		PTP1B = IC_50_ 9.40 ± 0.76 μM,	
Jejuguajavones D (**91**)		PTP1B = IC_50_ 35.94 ± 4.73 μM,	

**Table 9:** Minimal inhibitory concentration (MIC) values of *P. guajava* leaves extract.

Tested Microorganisms	Methanol	Ethanol	Acetone	Ethyl Acetate	Hot Water
*S. aureus*	10.3 ± 0.58 ^Da^	16.7 ± 1.15 ^Db^	29.3 ± 0.58 ^Ca^	41.7 ± 1.53 ^Ca^	7.0 ± 0.00 ^Db^
L. monocytogenes	8.3 ± 0.58 ^Da^	16.7 ± 1.15 ^Da^	14.3 ± 3.06 ^Da^	66.7 ± 5.8 ^Ba^	5.0 ± 0.00 ^Da^
*B. cereus*	10.3 ± 0.58 ^Da^	20.7 ± 1.52 ^Db^	12.3 ± 0.58 ^Da^	116.7 ± 15.28 ^Aa^	6.0 ± 0.00 ^Da^
S. typhmurium	5.0 ± 0.00 ^Db^	20.7 ± 1.52 ^Db^	10.3 ± 0.58 ^Db^	83.3 ± 2.89 ^Bc^	13.3 ± 0.58 ^Db^
*E. coli* O:157	7.3 ± 0.58 ^Db^	25.0 ± 0.00 ^Cb^	14.3 ± 3.06 ^Db^	100.0 ± 0.00 ^Ab^	14.3 ± 0.58 ^Db^
*P. mirabilis*	5.0 ± 0.00 ^Db^	20.7 ± 1.52 ^Db^	10.3 ± 0.58 ^Db^	116.7 ± 15.28 ^Ac^	13.3 ± 0.58 ^Db^
*P. aeruginosa*	8.3 ± 0.58 ^Db^	20.7 ± 1.52 ^Db^	8.3 ± 0.58 ^Db^	116.7 ± 15.28 ^Ac^	14.3 ± 0.58 ^Db^
*K. pneumoniae*	14.3 ± 3.06 ^Db^	29.3 ± 0.58 ^Ca^	10.3 ± 0.58 ^Db^	83.3 ± 2.89 ^Bb^	16.7 ± 1.15 ^Db^

Figures having same capital letters and small letters are significantly different at *p* = 0.05 probability by Fisher’s PLSD test. LSD 0.05 solvent 0.05 solvent 18.089, plant part 12.202.

## Data Availability

Not applicable.
